# Enhanced deep learning networks integrated by fractals for air quality index analysis

**DOI:** 10.3389/frai.2026.1892338

**Published:** 2026-08-20

**Authors:** S. Kala Nandhini, L. Thanga Mariappan

**Affiliations:** School of Computer Science Engineering and Information Systems, Vellore Institute of Technology, Vellore, Tamil Nadu, India

**Keywords:** air quality index, convolutional neural network, fractal dimension, fractal interpolation, long short-term memory

## Abstract

**Introduction:**

The Air Quality Index (AQI) provides daily information on the quality of outdoor air and increases with rising air emissions. Accurate AQI analysis and prediction are important for understanding and managing air pollution. This study develops an integrated deep learning framework incorporating a fractal approach to analyze and predict AQI.

**Methods:**

The developed framework consists of two main steps. First, AQI data are pre-processed using a fractal interpolation technique to address data discrepancies. Second, the pre-processed data are trained and tested using long short-term memory (LSTM), bidirectional long short-term memory (BiLSTM), and convolutional neural network with long short-term memory (CNN-LSTM) models for AQI prediction.

**Results:**

The proposed models are evaluated using AQI data from five major cities in India. Statistical performance metrics are used to assess and compare the predictive performance of the developed models.

**Discussion:**

The integration of fractal interpolation with deep learning provides a framework for handling discrepancies in AQI data and improving the analysis and prediction of air quality. The developed fractal-integrated deep learning models demonstrate their applicability for AQI prediction across the selected major Indian cities.

## 1 Introduction

As per the United Nations Environment Programme (UNEP), air pollution is one of the greatest risk factors for premature death. Air pollution causes approximately 8.1 million deaths every year, including premature deaths due to acute respiratory disease, heart disease, cancer, and stroke (Pozzer et al., 2020; Glencross et al., 2020; Ejaz et al., 2021). Therefore, to raise global awareness, the United Nations General Assembly has been celebrating the International Day of Clean Air for Blue Skies on 7^th^ September every year since 2019. Notably, air pollution and climate change are meticulously interconnected because they originate from common sources and heavily rely on greenhouse gas emissions. Addressing air pollution can bring significant benefits for all livelihoods on the earth, along with climate change. Realizing this critical situation, the concept of the Air Quality Index was designed and applied across the world to make pollution control decisions. The calculation of AQI integrates the concentrations of various air pollutants such as CO (Carbon Monoxide), SO2 (Sulfur Dioxide), NO2 (Nitrogen Dioxide), PM10 (Particulate Matters of less than 10 microns size), PM2.5 (Particulate Matter of less than 2.5 microns size), O3 (Ozone), Pb (Lead), NH3 (Ammonia), C6H6 (Benzene), BaP (Benzo(a)Pyrene), As (Arsenic), and Ni (Nickel) (Zhu et al., 2017; He et al., 2025a,b). For more information on air quality index and its connection with air pollution, visit (Tan et al., 2021; Xue et al., 2019; Haq, 2022; Horn and Dasgupta, 2024) and the references therein.

The air quality index quantitatively measures the quality of air and indicates its potential health effects. Moreover, it provides a rationale for eliminating the exposure of hazardous air pollutants and protecting public health from their adverse effects. In an effort to enhance the decision-making of regional environmental policy and prevent serious accidents due to air pollution, progressive air pollution predictions are crucial.

### 1.1 Retrospective studies

In this section, some recent notable deep learning and Machine Learning (ML) research works on the study of air pollution analysis and AQI prediction/forecasting are reviewed briefly. The article (Wu and Lin, 2019) has developed an optimal hybrid model based on secondary decomposition and Long Short Term Memory (LSTM) network for the AQI data series forecasting. Choosing wavelet decomposition as the primary decomposition technique, the proposed model is validated using the real AQI data of Beijing and Guilin. A hybrid model of LSTM-GRU is proposed to forecast the AQI for Particulate Matter (PM_2.5_) μ*m* and compared with several ML and deep learning models such as LSTM, GRU, and support vector machine. It is observed that the proposed hybrid model has exhibited better performance with R^2^ 0.84 and MAE 36.11 (Sarkar et al., 2022). For predicting the AQI of Ahmedabad city in India, several machine learning models, including SARIMA and LSTM, are compared in the study (Maltare and Vahora, 2023), and to handle the missing values, mean imputation is utilized. Comparing a variety of deep learning models like LSTM and ML models such as Huber Regressor, Adaptive Boosting, for AQI prediction, the study (Mishra and Gupta, 2024) has highlighted the performance of LSTM in accurately predicting the air quality. An optimized machine learning model is developed by combining Grey Wolf Optimization with the Decision Tree algorithm (Natarajan et al., 2024) and employed for AQI prediction in the major cities of India. The study (Mubeen et al., 2025) has employed artificial intelligence to improve air quality prediction and management by utilizing China's vast air quality monitoring data.

In addition to effectively utilizing current monitoring network data, the developed prediction and optimization approach provides precise future air quality forecasts, offering useful options for air pollution prevention. The research (Pande et al., 2024) has shown the outstanding performance of bidirectional recurrent neural network (RNN) in comparison with LSTM, Bidirectional Long Short Term Memory (BiLSTM), and Kernel Ridge Regression. The findings of AQI prediction for Delhi city using the Bi-RNN model resulted in R^2^ = 0.954. An integrated model of transformer encoder together with a BiLSTM network is trained and tested to predict the daily air quality index of three cities in China (Liu et al., 2025). The article (Nguyen et al., 2024) has proposed an attentive hybrid deep Quantum-inspired Particle Swarm Optimization model for predicting AQI and achieved a 19.03% reduction in MAE. Various ML models are compared for evaluating air quality grade and predicting daily air quality index in the article (Aram et al., 2024). The article (Özüpak et al., 2025) has investigated a comprehensive comparison of ten machine learning regression models in air quality forecasting. A hybrid Convolutional Neural Network–Long Short-Term Memory (CNN-LSTM) network has been utilized for fine-grained air pollution estimation in Zhang and Lam (2022). ANN and wavelet-ANN (WANN) models are used in Guo et al. (2023a) to forecast daily PM_2.5_ concentrations in Shanghai, while ANN and WANN models are employed in Guo et al. (2020) for short-term air pollution index forecasting. Both studies concluded that the WANN model outperformed the traditional ANN in terms of prediction accuracy. Using meteorological variables, an ANN model is developed in Guo et al. (2023b) to forecast hourly PM_2.5_ and PM_10_ concentrations; the Bayesian regularization (trainbr) approach produced the best prediction accuracy. The effectiveness of deep learning models for temperature prediction (Guo et al., 2023b, 2024a) and monthly climate prediction, including precipitation forecasting (Guo et al., 2024b), has been demonstrated using ANN, GRU, LSTM, CNN, CNN-GRU, and CNN-LSTM models. In Guo et al. (2025), ANN and LSTM models are investigated to predict ozone concentrations, with the LSTM model outperforming the ANN in terms of prediction accuracy. For other recent deep learning works on AQI forecasting and related studies, refer (He and Guo, 2024; Ravindiran et al., 2023; Rabie et al., 2024; Ahmed et al., 2024; Oezuepak and Aslan, 2024; Özüpak et al., 2026; Said et al., 2025).

From the aforementioned literature, though there are various ML and deep learning models employed for AQI prediction, data pre-processing technique like fractal interpolation is not utilized. In general, fractal functions are self-similar and highly irregular, hence better suited to reflecting the inherent complexity of real data (Băicoianu et al., 2024). In this study, the fractal interpolation function from the mathematical background is integrated with deep learning models as a novel method of pre-processing the AQI data. The literature (Gowrisankar et al., 2022; Kavitha et al., 2021) has acknowledged the role of fractal technique in pre-processing the real-world data like COVID-19 positive cases and greenhouse gas emissions. Therefore, in order to capture the complexity in the AQI data set and eliminate any missing information, this study employs the fractal interpolation technique. AQI data is collected from the five major cities of India, namely, Delhi, Faridabad, Bhopal, Jaipur, and Lucknow. The deep learning models, such as LSTM, BiLSTM, and CNN-LSTM models, are leveraged to predict pre-processed AQI and compare the results with the original non-pre-processed AQI data set. Finally, the predicted results of five cities are validated using the statistical evaluation metrics. Therefore, the novelty of this study lies in introducing a fractal interpolation-based pre-processing technique prior to predicting complex AQI time-series data using deep learning models. Consequently, the proposed approach preserves the hidden nonlinear structures, multiscale variability, and intrinsic complexity present in environmental data. The key contributions of this study are given in the following:

- To handle missing data and capture the intrinsic complexity and irregular behavior of air quality dynamics, a fractal interpolation function is employed as a pre-processing technique. Furthermore, the fractal dimension of the pre-processed AQI time series is estimated to quantify the complexity and self-similar characteristics of the data.
- Three fractal-integrated deep learning architectures—LSTM, BiLSTM, and CNN-LSTM—are developed to predict pre-processed AQI data across five major Indian cities. The proposed structure effectively captures the multiscale, non-linear, and temporal features present in AQI time-series data by combining sequential deep neural networks with fractal-based pre-processing techniques.
- The proposed fractal-based models considerably improve prediction performance over models trained on raw AQI data, as demonstrated using a comprehensive comparative investigation. The superiority and predictive reliability of the fractal-integrated models are confirmed by statistical performance metrics, which quantitatively validate the efficacy and robustness of the developed approaches.

The organization of the remaining sections is as follows. Section 2 presents the methods and materials required for this study, including the selection of AQI data. An algorithm associated with the fractal integrated deep learning models is provided in Section 3. Section 4 is devoted to exploring and comparing the prediction results of raw AQI data and fractal pre-processed AQI data using LSTM, BiLSTM, and CNN-LSTM networks. Providing the limitations in Section 5, the study ends with the Conclusion in Section 6.

## 2 Methods and materials

### 2.1 Long short-term memory deep learning network

**Figure 1:**
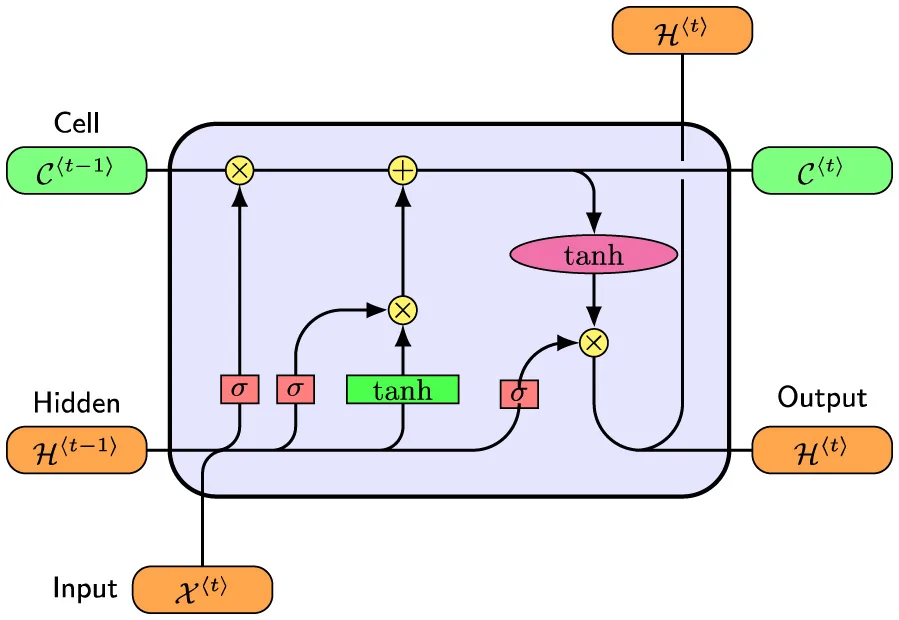
Architecture of long short term memory.

Hochreiter and Schmidhuber (1997) developed the LSTM network to effectively store past information while retaining the recent states' significance. The simple internal architecture of the LSTM is presented in Figure 1.

A standard LSTM model comprises a cell and three gates, namely forget gate, input gate, and output gate. The forget gate ${ℱ}_{t}$ reads the previous hidden state $H_{t-1}$ and the current input $X_{t}$ to produce an output value between 0 and 1 using sigmoid activation function. It is mathematically expressed using,


$$\begin{array}{l} F_{t}=\sigma \left(W_{F}\cdot \left[H_{t-1},X_{t}\right]+B_{f}\right), \end{array}$$

where $W_{F}$ are weights, $B_{F}$ are bias vectors, and σ is the sigmoid function.

The value of updating the memory state from the input is determined by the input gate *i*_*t*_. The candidate cell state $\hat{C}$ employs a tanh function to determine what data should be used for calculating the cell state $C_{t}$, whereas the input gate uses a sigmoid function. The states *i*_*t*_ and ${\hat{C}}_{t}$ are respectively given by,


$$\begin{array}{l} i_{t}=\sigma \left(W_{i}\cdot \left[H_{t-1},X_{t-1}\right]+B_{i}\right),\\ {\hat{C}}_{t}=\tanh\left(W_{c}\cdot \left[H_{t-1},X_{t-1}\right]+B_{c}\right), \end{array}$$

where tanh is the hyperbolic tangent activation function, which outputs a value between −1 and 1.

Combining the values of forget gate, input gate, candidate cell state, and previous cell state $C_{t-1}$, cell state information is updated. The updated cell state is given by


$$\begin{array}{l} C_{t}=F_{t}\cdot C_{t-1}+i_{t}\cdot {\hat{C}}_{t}, \end{array}$$

The output gate $O_{t}$ determines how the cell state will affect the current output using the previous hidden state and current input. The corresponding expressions are given by


$$\begin{array}{l} O_{t}=\sigma \left(W_{C}\cdot \left[H_{t-1},X_{t}\right]+B_{O}\right),\\ H_{t}=O_{t}\cdot \tanh\left(C_{t}\right). \end{array}$$

Several problems pertaining to sequential models with dependencies, such as the case of AQI and weather forecasting, can be resolved attributable to the gate structure employed by LSTM networks.

### 2.2 Bidirectional LSTM deep learning network

**Figure 2:**
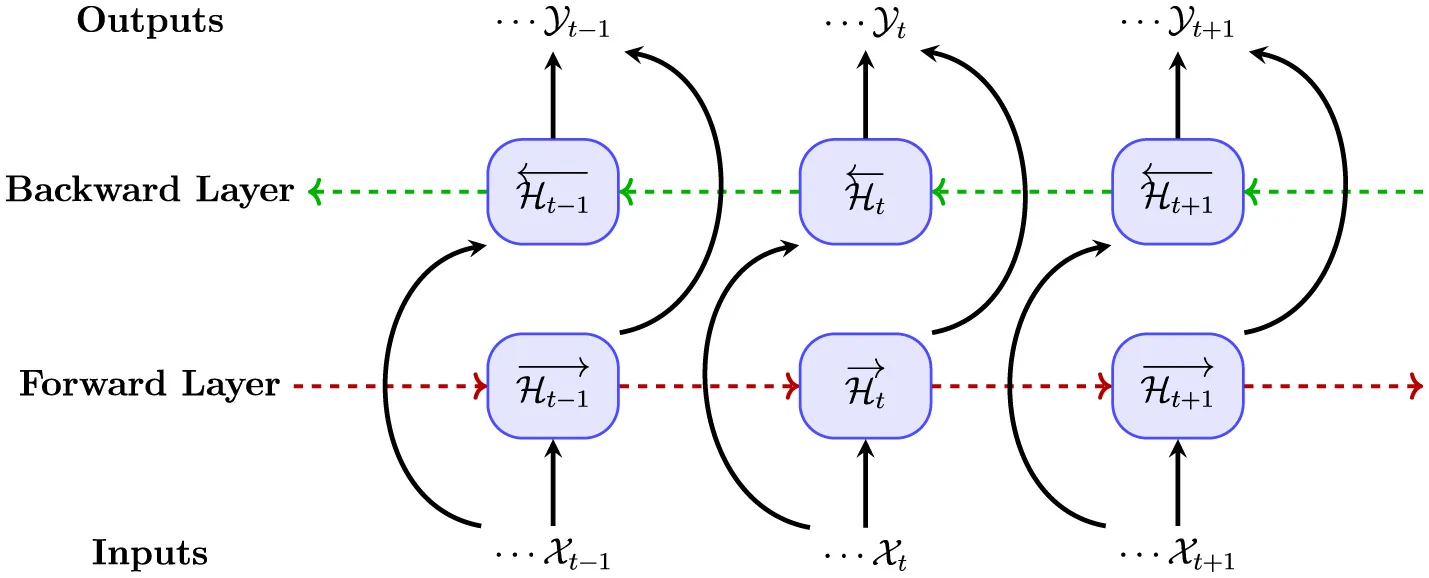
Architecture of bidirectional long short term memory.

A unique RNN introduced to process the data sequences in both directions is the bidirectional recurrent neural network. The fundamental idea is to process the data sequence in two different ways: first, in the standard forward direction, and second, in the backward direction. As a consequence, RNNs expand their capabilities to circumstances where information from the past and future impacts the outcomes. The concept of bidirectional RNN is combined with LSTM to create the “BiLSTM” architecture. BiLSTM is implemented with two LSTM layers, as demonstrated in Figure 2. The horizontal dotted arrows indicate the bidirectional flow between each unit, vertical solid arrows indicate unidirectional flow from the input layer to the output layer via the hidden layer, and the curved arrows represent the forward and backward unit flows. For more information on the BiLSTM structure, see Zhao et al. (2018).

A single BiLSTM layer is concatenated using a forward sequence and a backward sequence, which is shown in the following expressions:


$$\begin{array}{l} \vec{H_{t}}=LSTM\left(X_{t},\vec{H_{t-1}}\right)\\ \overset{⃖}{H_{t}}=LSTM\left(X_{t},\overset{⃖}{H_{t-1}}\right)\\ Y_{t}=g\left(W_{\vec{H}Y}\overset{⃖}{H_{t}}+W_{\overset{⃖}{H}Y}\overset{⃖}{H_{t}}+B_{Y}\right) \end{array}$$

where *g*(·) denotes the activation function, $W_{ij}$ denotes the weight from *i* to *j*, and $B_{j}$ denotes the bias at *j*th layer. BiLSTM processes the data flow in both directions, allowing for a better learning process than LSTM, which often only permits inputs in one direction.

### 2.3 CNN-LSTM network

CNNs are mostly used for computer vision tasks, where they extract high-level features by convolving filters on the input data (such as an image). CNNs are able to identify both local and global patterns in the data because of their hierarchical structure. CNN architecture comprises three layers: a convolution layer, a pooling layer, and a fully connected layer. By passing a filter (kernel) of a specific size across the output of the preceding layer, the convolution layer implements the linear process. The main activation function that is frequently employed with CNN is the non-linear ReLu function in order to raise the degree of non-linearity and set all negative values in the feature map to zero. In the combined CNN-LSTM architecture, CNN layers are used to extract features from the input data. These features are altered and fed into LSTM layers after being retrieved. This combination enables the model to use CNNs to find local patterns or features in the input data and then use LSTMs to simulate the temporal aspects of these features.

### 2.4 Fractal interpolation technique

When investigating the real-world data set, common problems include the data being incomplete, containing missing information, or being highly irregular. Fractal interpolation is a method to address such unstructured data. In this subsection, we recollect the construction of fractal interpolation for further implementation with deep learning models. A special kind of Iterated Function System (IFS) generates the fractal interpolation function, the prescribed dataset size can be enlarged by increasing the iteration step. Consider the frequency data *f*_0_, *f*_1_, …, *f*_*N*_ for the prescribed time *t*_0_, *t*_1_, …, *t*_*N*_. Then the desired interpolation data set is formed by $\left\{\left(t_{k},f_{k}\right)\in {\mathbb{R}}^{2}:k=0,1,\ldots ,N\right\},\;N\geq 2$ with *t*_0_ < *t*_1_ < ⋯ < *t*_*N*_. The affine transformations for constructing the fractal interpolation function are given by


$$\begin{array}{l} w_{k}\left(\text{X}\right)=\left[\begin{array}{cc} a_{k} & 0\\ c_{k} & \alpha_{k} \end{array}\right]\text{X}+\left[\begin{array}{c} b_{k}\\ d_{k} \end{array}\right],\;\text{X}=\left[\begin{array}{c} t\\ f \end{array}\right]. \end{array}$$ (1)

obeying the conditions *w*_*k*_(*t*_0_, *f*_0_) = (*t*_*k*−1_, *f*_*k*−1_), *w*_*k*_(*t*_*N*_, *f*_*N*_) = (*t*_*k*_, *f*_*k*_), *k* = 1, 2, …, *N*, where *a*_*k*_, *b*_*k*_, *c*_*k*_, *d*_*k*_ and α_*k*_ are all real parameters. The system of equation given in Equation 1 with end point conditions has a unique solution when α_*k*_ is predefined. Further, the constants *a*_*k*_, *b*_*k*_, *c*_*k*_, *d*_*k*_ can be obtained uniquely in terms of α_*k*_ as follows.


$$\begin{array}{l} a_{k}=\frac{t_{k}-t_{k-1}}{t_{N}-t_{0}}\\ b_{k}=\frac{t_{N}t_{k-1}-t_{0}t_{k}}{t_{N}-t_{0}}\\ c_{k}=\frac{\left(f_{k}-f_{k-1}\right)-\alpha_{k}\left(f_{N}-f_{0}\right)}{t_{N}-t_{0}}\\ d_{k}=\frac{\left(t_{N}f_{k-1}-t_{0}f_{k}\right)-\alpha_{k}\left(t_{N}f_{0}-t_{0}f_{N}\right)}{t_{N}-t_{0}} \end{array}$$ (2)

for *k* = 1, 2.…, *N*. The predefined parameter |α_*k*_| < 1 is known as the vertical scaling factor, which highly influences the structure of the fractal interpolation function. If 0 ≤ α_*k*_ < 1 with $\overset{N}{\underset{k=1}{\sum }}|\alpha_{k}|>1$ and the interpolation points are not collinear, then the fractal dimension of the fractal function is the unique solution *D* of the following equation:


$$\begin{array}{l} \overset{N}{\underset{k=1}{\sum }}|\alpha_{k}|a_{k}^{D-1}=1. \end{array}$$ (3)

Otherwise the fractal dimension is one. In this study, the scaling parameter α_*k*_ is optimally chosen as


$$\begin{array}{l} \alpha_{k}=\frac{f_{k}-f_{k-1}}{\sqrt{{\left(\max_{k}\left(f_{k}\right)-\min_{k}\left(f_{k}\right)\right)}^{2}+{\left(f_{k}-f_{k-1}\right)}^{2}}} \end{array}$$ (4)

for *k* ∈ {1, 2, …, *N*}. Unlike standard pre-processing techniques such as linear interpolation, polynomial interpolation, or smoothing-based approaches, fractal interpolation is capable of preserving the natural irregularity and nonlinear fluctuations contained in AQI time-series data. Air quality measurements often reveal complicated fluctuations due to the combined influence of meteorological conditions, emission sources, and seasonal trends. Traditional interpolation methods often rely on global assumptions about data trends and may suppress local variations, whereas fractal interpolation creates new data points by leveraging self-similarity features and local scaling connections. Therefore, fractal interpolation is employed in this study as a pre-processing approach to keep the complex temporal patterns of AQI data while enhancing data resolution.

### 2.5 Evaluation metrics

For the present study, six metrics are used for evaluating the performance of the proposed fractal integrated deep learning models. The errors of the predicted AQI data obtained using LSTM and BiLSTM are assessed using the metrics RMSE, NRMSE, MAE, MAPE, NMAPE, and R^2^. The formulas of RMSE, NRMSE, MAE, MAPE, NMAPE, and R^2^ are given by


$$\begin{array}{l} \text{RMSE}=\sqrt{\frac{\sum_{t=1}^{N}{\left(f_{t}-{\hat{f}}_{t}\right)}^{2}}{N}} \end{array}$$

$$\begin{array}{c} \text{NRMSE }=\frac{\sqrt{\frac{1}{N}\sum_{t=1}^{N}{\left(f_{t}-{\hat{f}}_{t}\right)}^{2}}}{\frac{1}{N}\sum_{t=1}^{N}f_{t}}\\ \text{MAE }=\frac{1}{N}\sum_{t=1}^{N}\left|f_{t}-{\hat{f}}_{t}\right|\\ \text{MAPE }(\%)=\frac{1}{N}\sum_{t=1}^{N}\left|{\hat{f}}_{t}-f_{t})\right|\;\times 100\%\\ \text{NMAPE }(\%)\;=\frac{1}{N}\sum_{t=1}^{N}\left|\frac{f_{t}-{\hat{f}}_{t})}{\frac{1}{N}\sum_{t=1}^{N}f_{t})}\right|\;\times 100\%\\ R^{2}\;=1-\frac{\sum_{t=1}^{N}{\left(f_{t}-{\hat{f}}_{t}\right)}^{2}}{\sum_{t=1}^{N}{\left(f_{t}-\bar{f}\right)}^{2}}. \end{array}$$

Here, with respect time *t*, *f*_*t*_ represents the original value (original frequency) and $\hat{f}\left(t\right)$ represents the predicted value (predicted frequency), for *t* = 1, 2, …, *N*. The average of *f*_*t*_ is denoted as $\bar{f}$. Each metric value depicts the disparity between the original AQI and predicted AQI. It is well known that performance improves with a lower metric value.

Algorithm 1 Deep learning algorithm with fractal based AQI Prediction. 
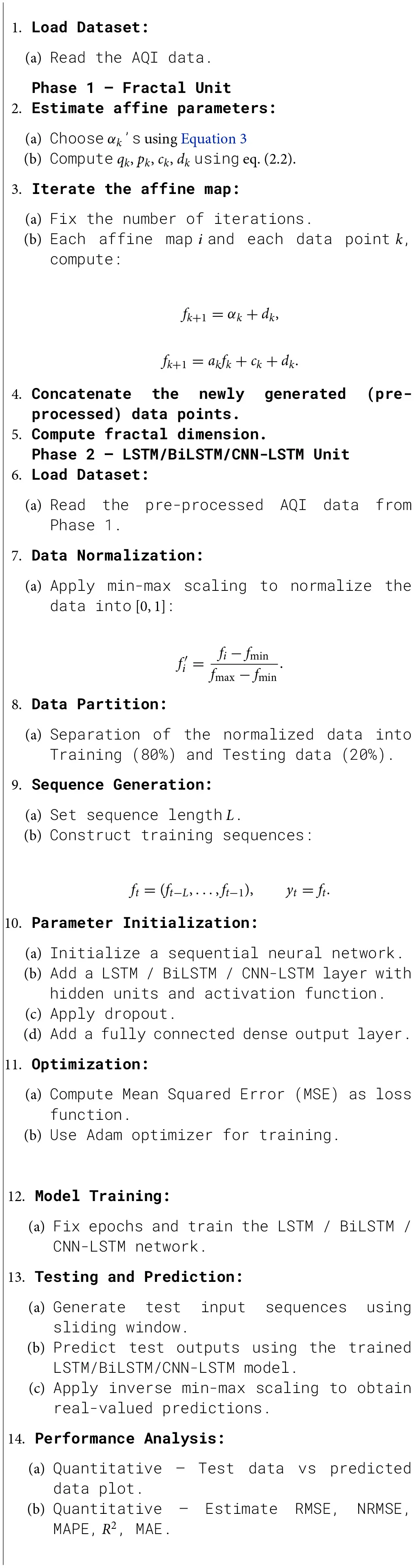

### 2.6 Data description

In this study, the AQI data is collected from the Central Pollution Control Board (CPCB) (Central Pollution Control Board, CPCB). The CPCB functions under the Ministry of Environment, Forest and Climate Change, Government of India. Enhancing the nation's air quality and preventing, controlling, or reducing air pollution are two of the CPCB's main responsibilities.

**Figure 3:**
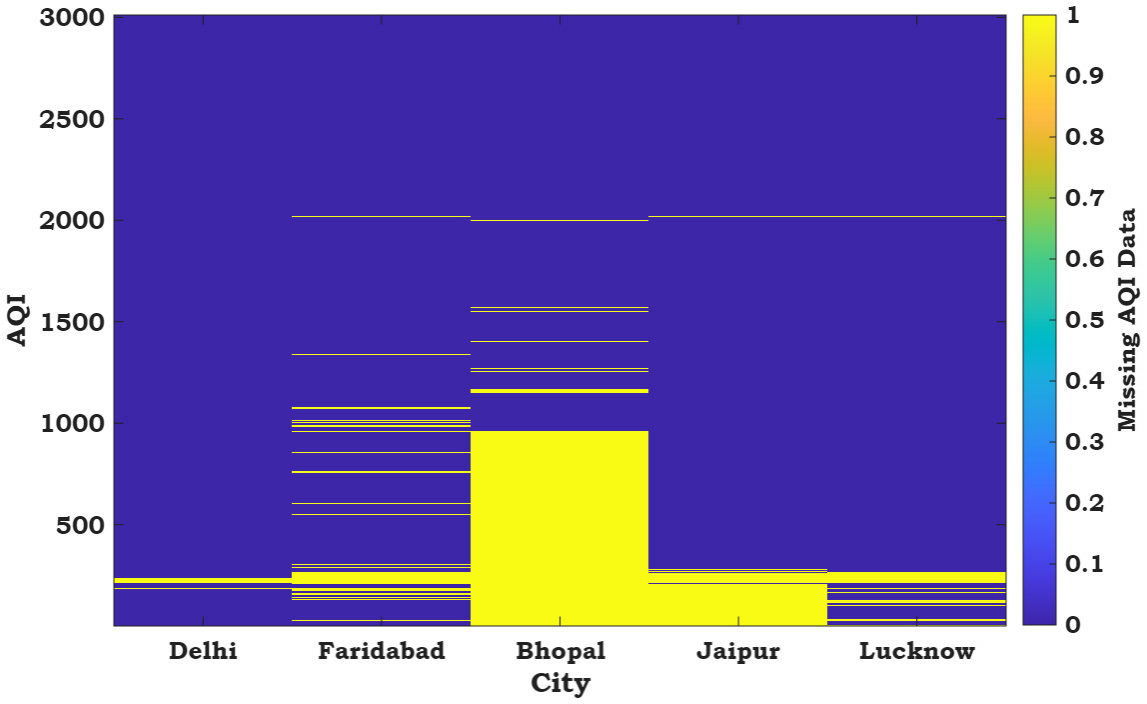
Heat map for missing AQI values.

**Figure 4:**
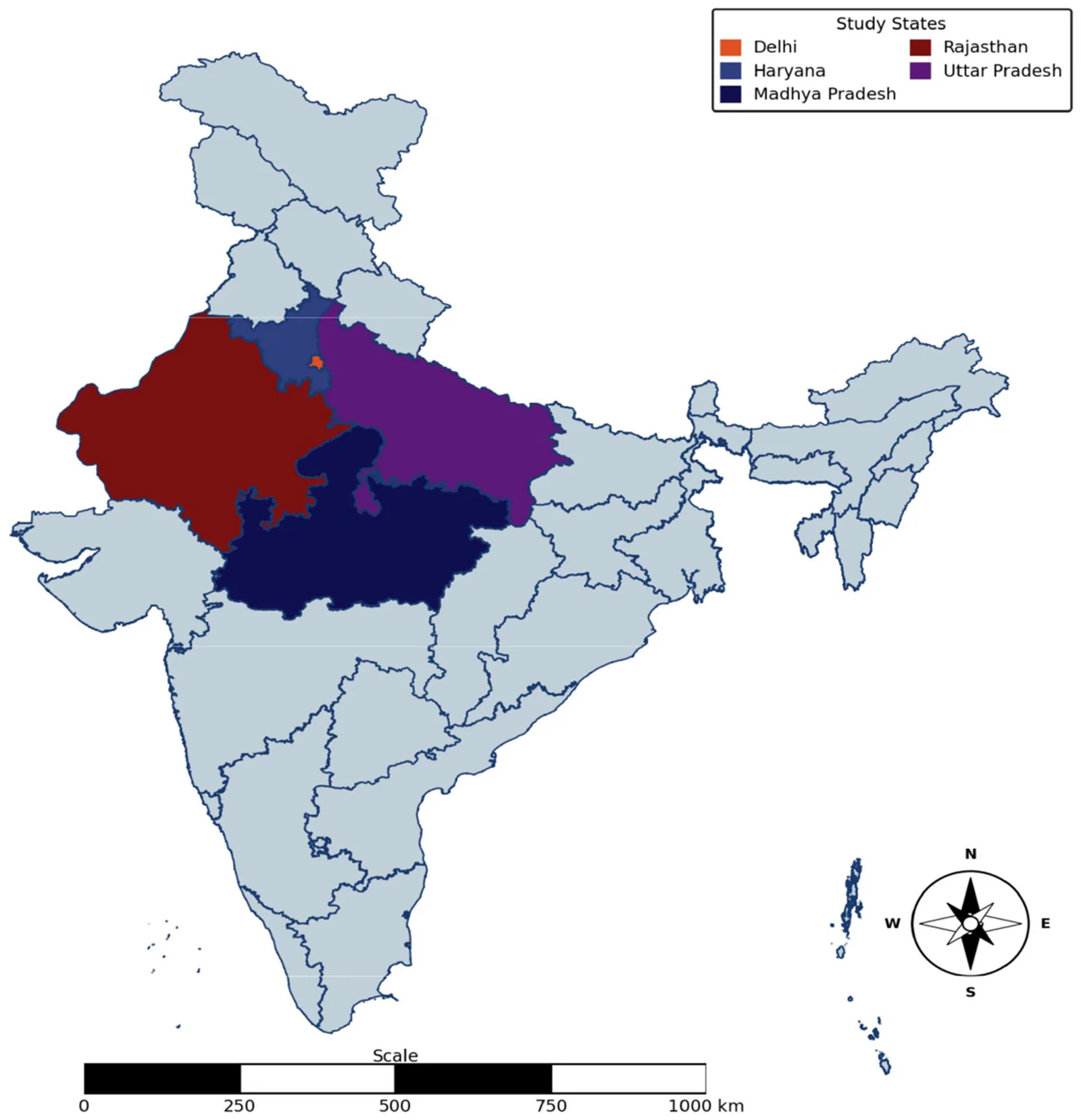
Location map of study region in India

Study region: The daily air quality index of Delhi is collected from January 2017 to March, 2025. The data set includes 3,011 samples (in days). The heat map of the samples with missing data is presented in Figure 3. In order to focus on the air quality of Delhi, the capital of India, the daily AQI data is collected from its neighboring states major cities, namely, Faridabad (Haryana), Bhopal (Madhya Pradesh), Jaipur (Rajasthan), and Lucknow (Uttar Pradesh). The study region is displayed in Figure 4.

As per the Indian National Air Quality Standards (INAQS), there are 12 air pollutant parameters notified by CPCB to estimate the AQI in India. The air pollutants are: CO (Carbon Monoxide), SO2 (Sulfur Dioxide), NO2 (Nitrogen Dioxide), PM10 (Particulate Matters of less than 10 microns size), PM2.5 (Particulate Matter of less than 2.5 microns size), O3 (Ozone), Pb (Lead), NH3 (Ammonia), C6H6 (Benzene), BaP (Benzo(a)Pyrene), As (Arsenic), and Ni (Nickel). The following two steps are used to calculate the AQI in India: (i) computing the sub-index (*I*_*i*_) for each air pollutant (*X*_*i*_) from its actual air pollution concentration, (ii) applying the aggregation function (*F*) on the sub-index collection to get the final AQI. In order to avoid ambiguity and eclipsing, the following maximum function is used for calculating AQI in India:


$$AQI=\max\left\{I_{1},I_{2},\ldots ,I_{n}\right\}.$$

The AQI range-based description of air quality level, and its associated health effects, is given in Table 1. In Table 1, Good gives the good air quality and minimal health impact. Satisfactory represents less air pollution and creates minor breathing discomfort to sensitive people. Moderate means sensible air pollution, which causes breathing disturbances to people with existing asthma, lung problems, and heart diseases. Poor means significant air pollution, and it affects most people when exposed for a prolonged period. Very Poor means intensified air pollution, which creates respiratory illness on continued exposure. Severe means vulnerable air pollution and affects healthy individuals, and has an extreme impact on those who are suffering from illness.

**Table 1:** Classification of air quality index in India.

Range of AQI	Category	Color code
0–50	Good	
51–100	Satisfactory	
201–300	Poor	
301–400	Very Poor	
401–500	Severe	

The datasets consist of chronological AQI observations indicating the temporal change of air pollution levels. Before model development, the datasets were subjected to preprocessing methods, including handling missing values, standardization, and fractal interpolation-based data augmentation. The fractal interpolation approach creates additional data points while keeping the essential properties of the original AQI series, hence boosting the ability of deep learning models to learn complex nonlinear patterns.

## 3 Proposed model

**Figure 5:**
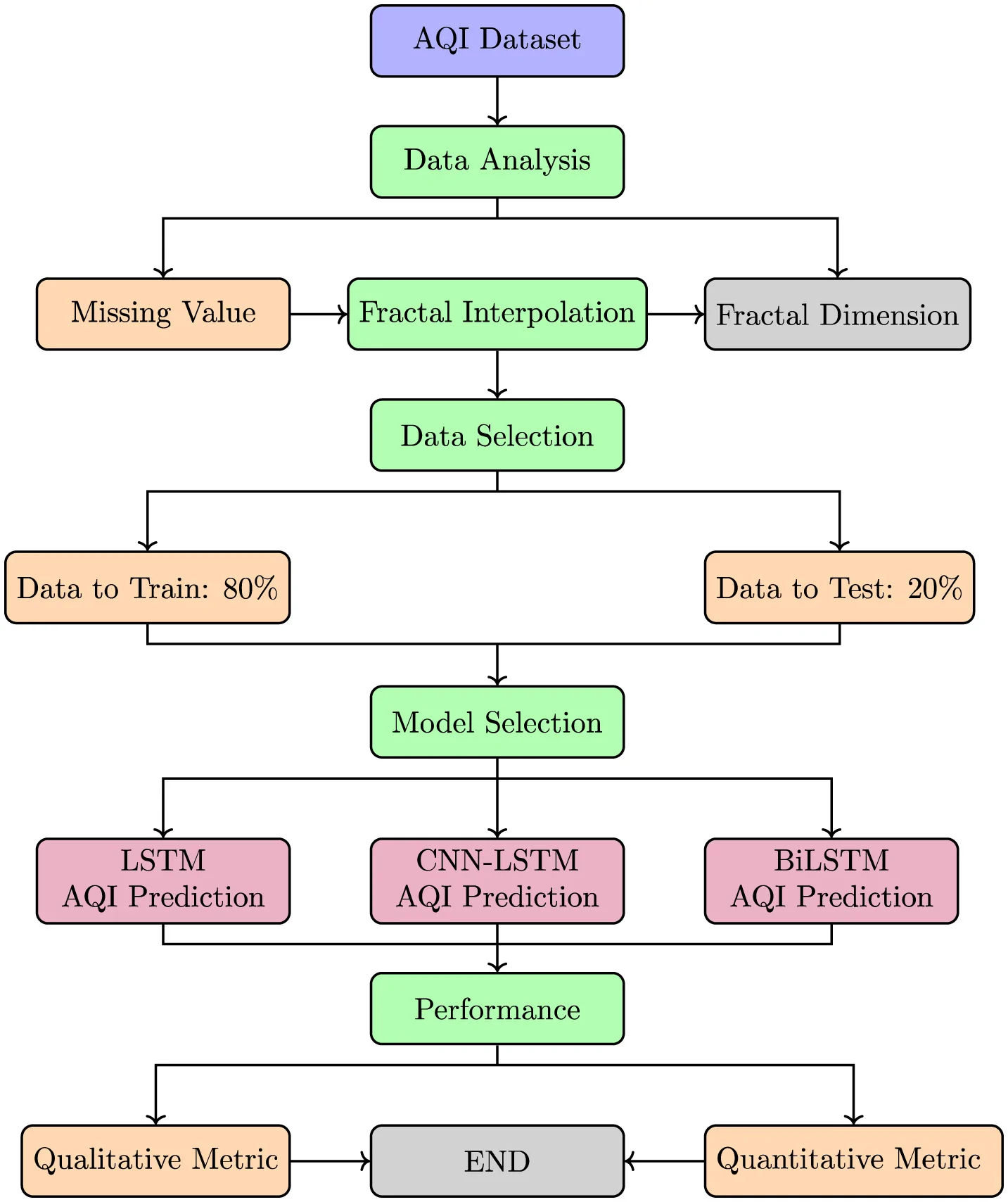
Architecture of the proposed deep learning network with fractals framework for AQI prediction.

This section presents the proposed fractal integrated deep learning models via an algorithm. The algorithm consists of two phases. In the first phase, AQI data is pre-processed using the fractal interpolation technique discussed in section 2.3, and the fractal dimension is estimated for the fractal pre-processed AQI data. In the second phase, pre-processed AQI data is predicted using either an LSTM, a BiLSTM, or a CNN-LSTM network, and the results are tested using statistical metrics. The architecture of the proposed deep learning network with a fractals framework for AQI prediction is provided in Figure 5.

## 4 Results and discussion

**Figure 6:**
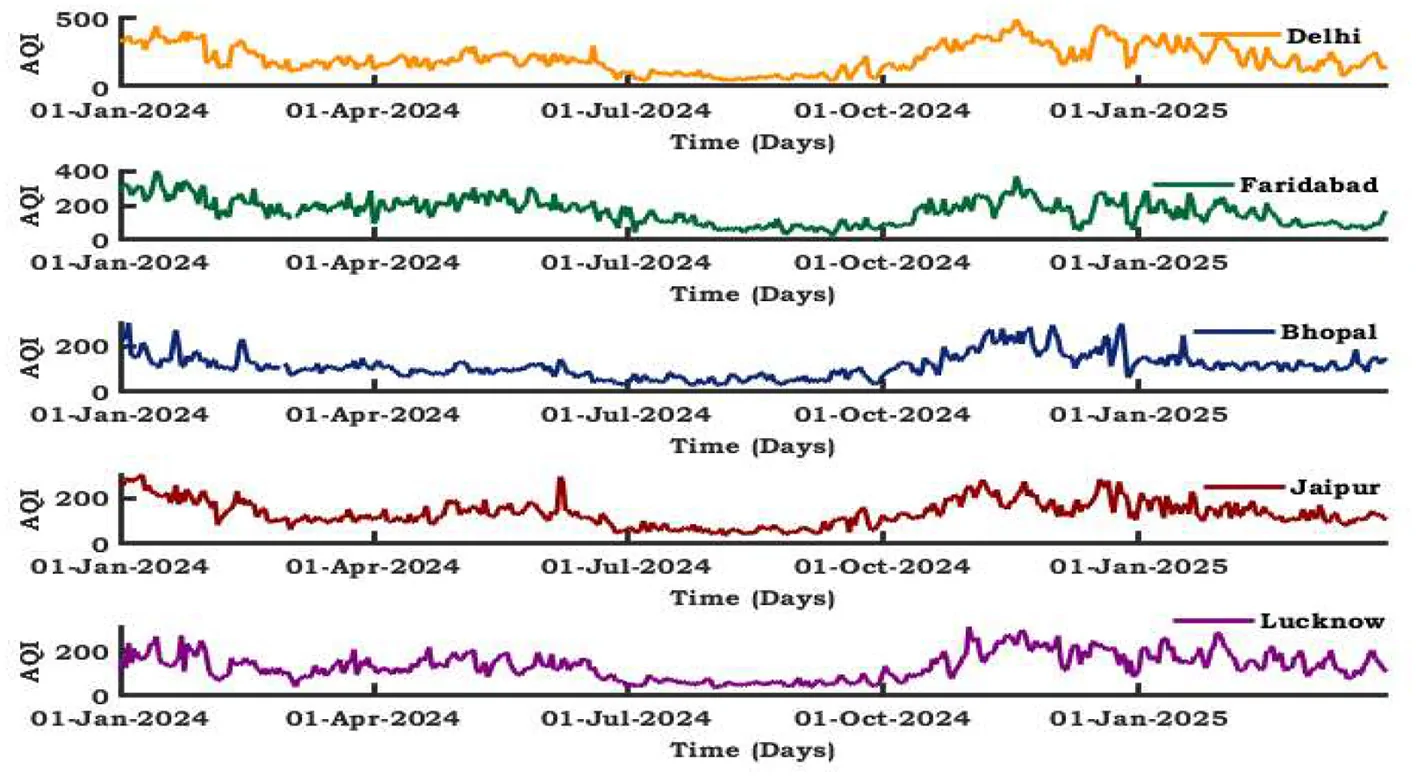
Daily AQI variation across cities (January 2024–March 2025).

This section clearly describes the following: data pre-processing using fractal interpolation, advantages of the fractal technique in pre-processing, implementation of LSTM, BiLSTM, and CNN-LSTM models, and comparison of deep learning models with the use of original and pre-processed AQI data. In this work, the air quality index of five Indian cities, namely, Delhi, Faridabad, Bhopal, Jaipur, and Lucknow, is studied. Figure 6 illustrates the graphical representation of original AQI data of five cities from January 2024 to March 2025. Among the five cities, the AQI is higher for Delhi than the other four cities on average.

### 4.1 Fractal pre-processing

**Figure 7:**
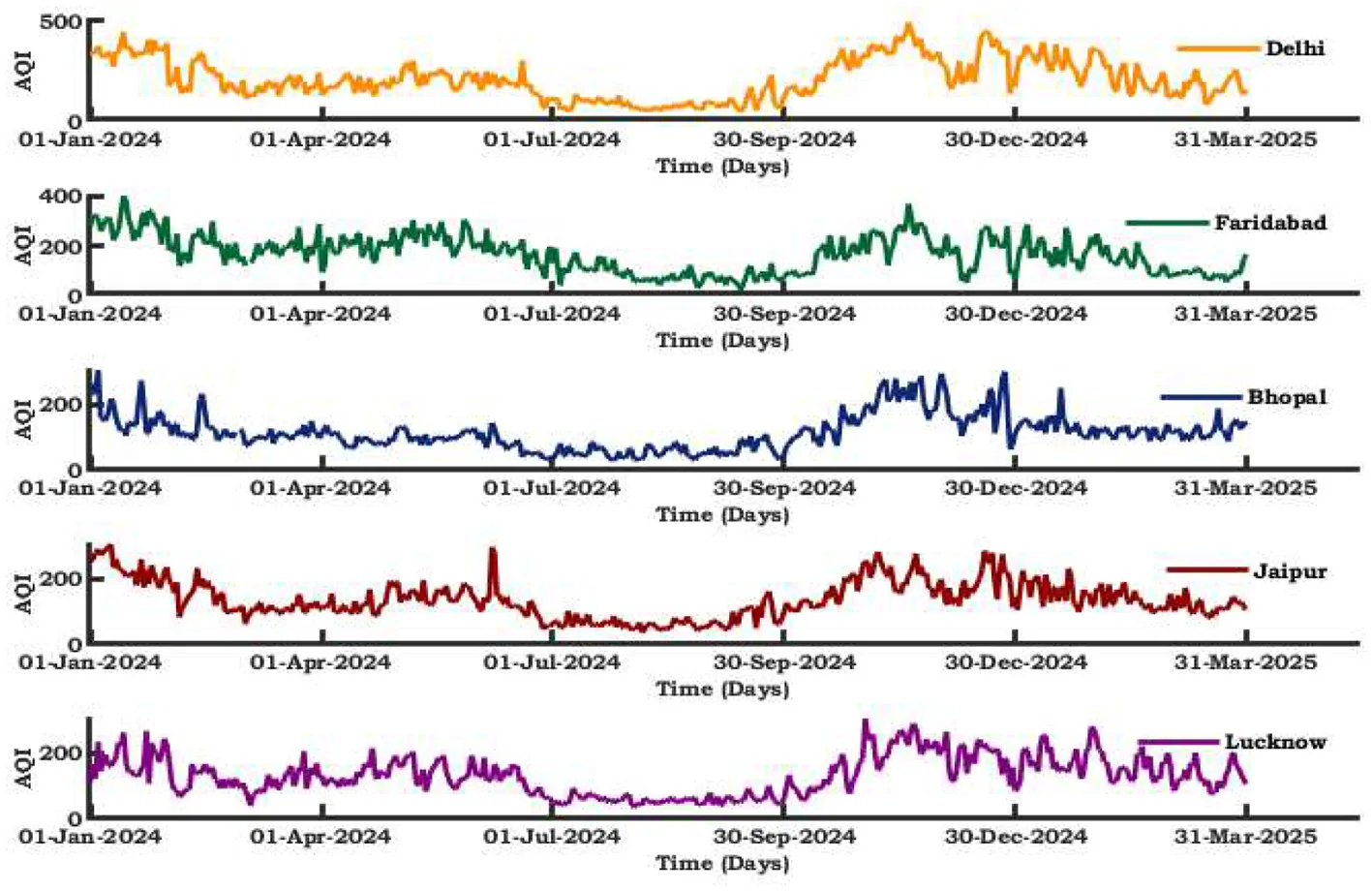
Pre-processed daily AQI variation across cities (January 2024–March 2025).

Fractal interpolation function generates a huge number of data points within the given finite data set, as it is generated using the iterated function system. For *k* + 1 number of sample points, the fractal function generates a total number of *k*^*n*+1^ + 1 data points at the end of *n*th iteration. The process of pre-processing using the fractal function omits the problem of missing data as much as possible. Further, it works effectively for a data set showing any irregularity with high fluctuations, thus more beneficial for any real-world data like AQI. In this study, 3,011 sample points are increased to a million by taking an iteration equal to 2. Due to the self-similar nature of the fractal function, irregularity is preserved between each 2 data points. The pre-processed AQI data using fractal interpolation (with iteration = 2) is illustrated in Figure 7. The complexity of original AQI data in Figure 6 is exactly preserved in Figure 7 with a huge number of data points. In addition, the fractal dimension of pre-processed AQI is estimated using Equation 4 for all five cities, and the results are depicted in Table 2. The obtained non-integer fractal dimensions indicate the irregular distribution of AQI throughout the selected time duration from Jan 2024 to Dec 2024. The lesser fractal dimension of Delhi (1.3331) clarifies that though the peak of AQI is around 500, its distribution is less irregular than the other cities. Meanwhile, with 200 AQI values, Bhopal, Jaipur, and Lucknow have higher fractal dimensions, indicating more complexity in their distribution than Delhi.

**Table 2:** Fractal dimension of pre-processed AQI.

Study regions	Fractal dimension
Delhi	1.3331
Faridabad	1.3452
Bhopal	1.3712
Jaipur	1.3757
Lucknow	1.3745

Figures 6, 7 clearly elucidate the self-similar pattern and periodic behavior of the daily AQI variation across the selected cities. In particular, AQI levels are higher at the beginning and end of the year, whereas relatively lower during the middle of the year. This same pattern follows throughout the selected period with fluctuations. In order to understand the AQI pattern throughout the selected period, the data from January 2024 to December 2024 is pre-processed using the fractal interpolation. Further, their self-similar nature is explored from the recurring pattern across time scales, and the non-integer fractal dimension confirms the fractal characteristic of the AQI pattern. Hence, although the dataset spans January 2017 to March 2025, fractal analysis relies on the self-similar nature of the system over appropriate scales, which is often locally valid rather than globally uniform in real-world data. In addition, the same period is employed for the prediction of the AQI recent trend using deep learning networks.

### 4.2 Implementation of deep learning models

LSTM, BiLSTM, and CNN-LSTM models are implemented using the TensorFlow Keras library in the Python programming environment. The LSTM model has a single LSTM layer with 100 neurons and a tanh activation and one Dense layer with a single neuron and a linear activation. The provided data is split into 80% data sample that is trained, and 20% of data sample that is tested with a batch size of 32. The LSTM model is trained and tested for 50 epochs. Next, for the BiLSTM model, a bidirectional LSTM layer with 100 neurons and tanh activation is employed, and a Dense layer is fixed with one neuron and linear activation to produce a single variable output. Batch size is set to 32, and the training is conducted for 50 epochs. In both models, a dropout function with 0.2 is utilized to avoid data overfitting. The layer details and training configuration of LSTM and BiLSTM are provided in Table 3. The CNN model consists of a 1D convolutional layer with 64 filters, kernel size 2, and ReLU activation function, followed by a max-pooling layer with pool size 2 to reduce dimensionality and extract significant features. In order to understand long-term sequential dependencies, the collected features are flattened and passed to an LSTM layer with 100 neurons with tanh activation. To avoid overfitting, a dropout layer with a dropout rate of 0.2 is included. Lastly, the output prediction is generated using a Dense layer with a single neuron.

**Table 3:** Layer details and training configuration of deep learning models.

LSTM model			BiLSTM model		
Layer type	Quantity	Activation	Layer type	Quantity	Activation
LSTM	100	tanh	BiLSTM	100	tanh
Dropout	0.2	–	Dropout	0.2	–
Dense	1	linear	Dense	1	Linear
Sequence length	10	–	Sequence length	10	–
Optimizer	Adam	–	Optimizer	Adam	–
Epochs	50	–	Epochs	50	–

### 4.3 Prediction results and performance evaluation

**Figure 8:**
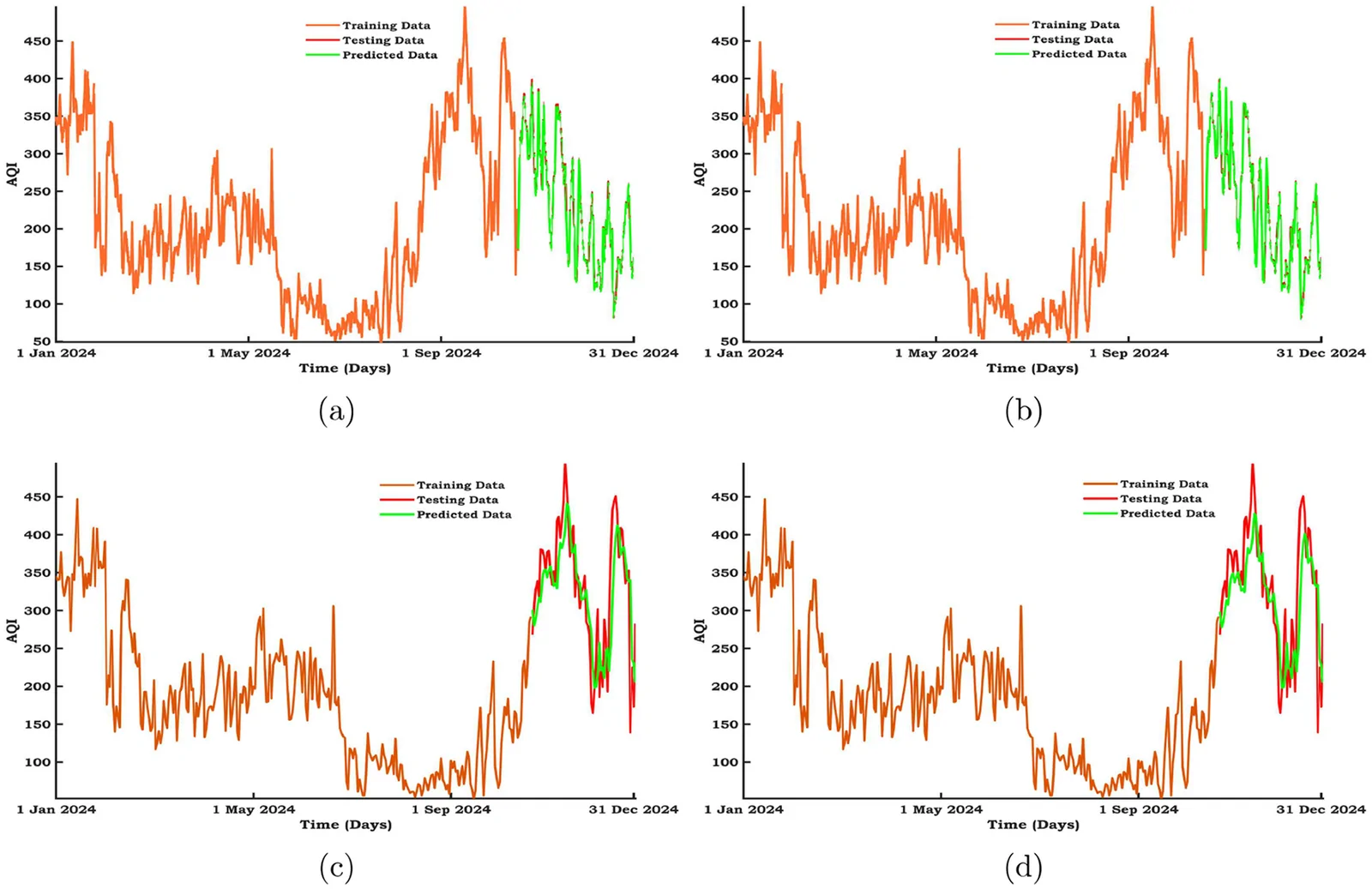
Comparison of AQI data associated with Delhi after & before fractal pre-processing: **(a)** F-LSTM, **(b)** F-BiLSTM, **(c)** LSTM, **(d)** BiLSTM.

**Figure 9:**
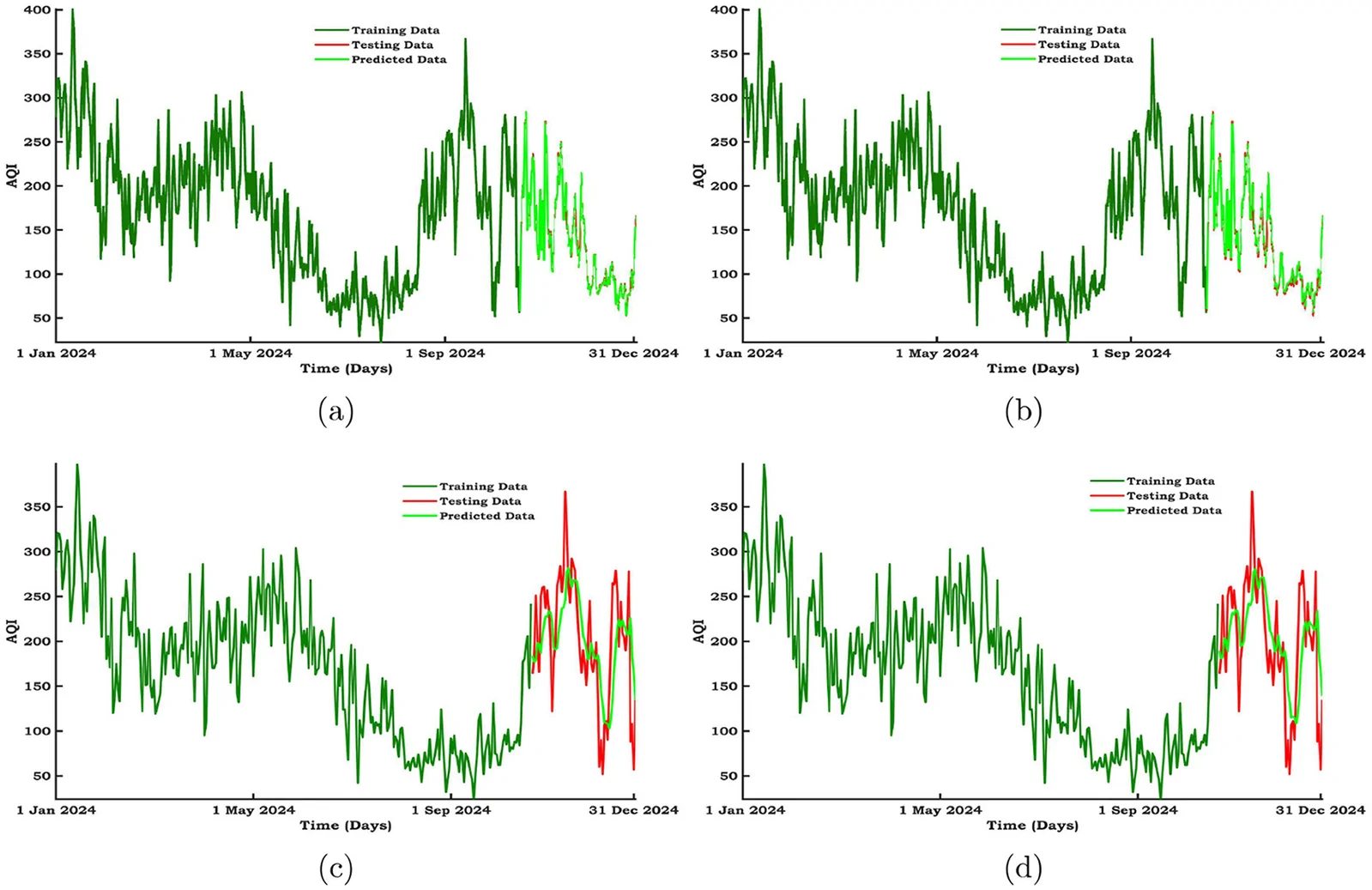
Comparison of AQI data associated with Faridabad after & before fractal pre-processing: **(a)** F-LSTM, **(b)** F-BiLSTM, **(c)** LSTM, **(d)** BiLSTM.

**Figure 10:**
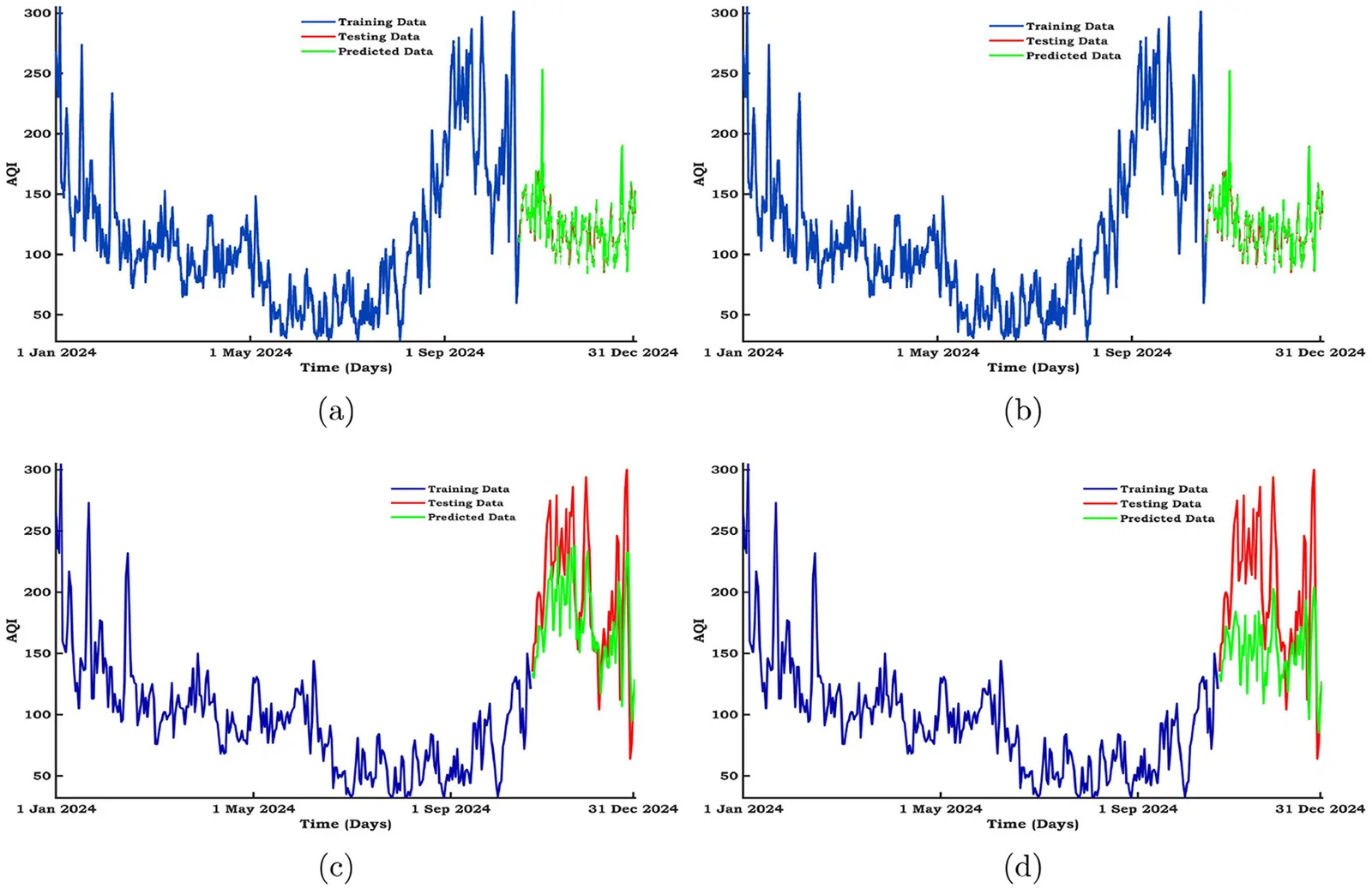
Comparison of AQI data associated with Bhopal after & before fractal pre-processing: **(a)** F-LSTM, **(b)** F-BiLSTM, **(c)** LSTM, **(d)** BiLSTM.

**Figure 11:**
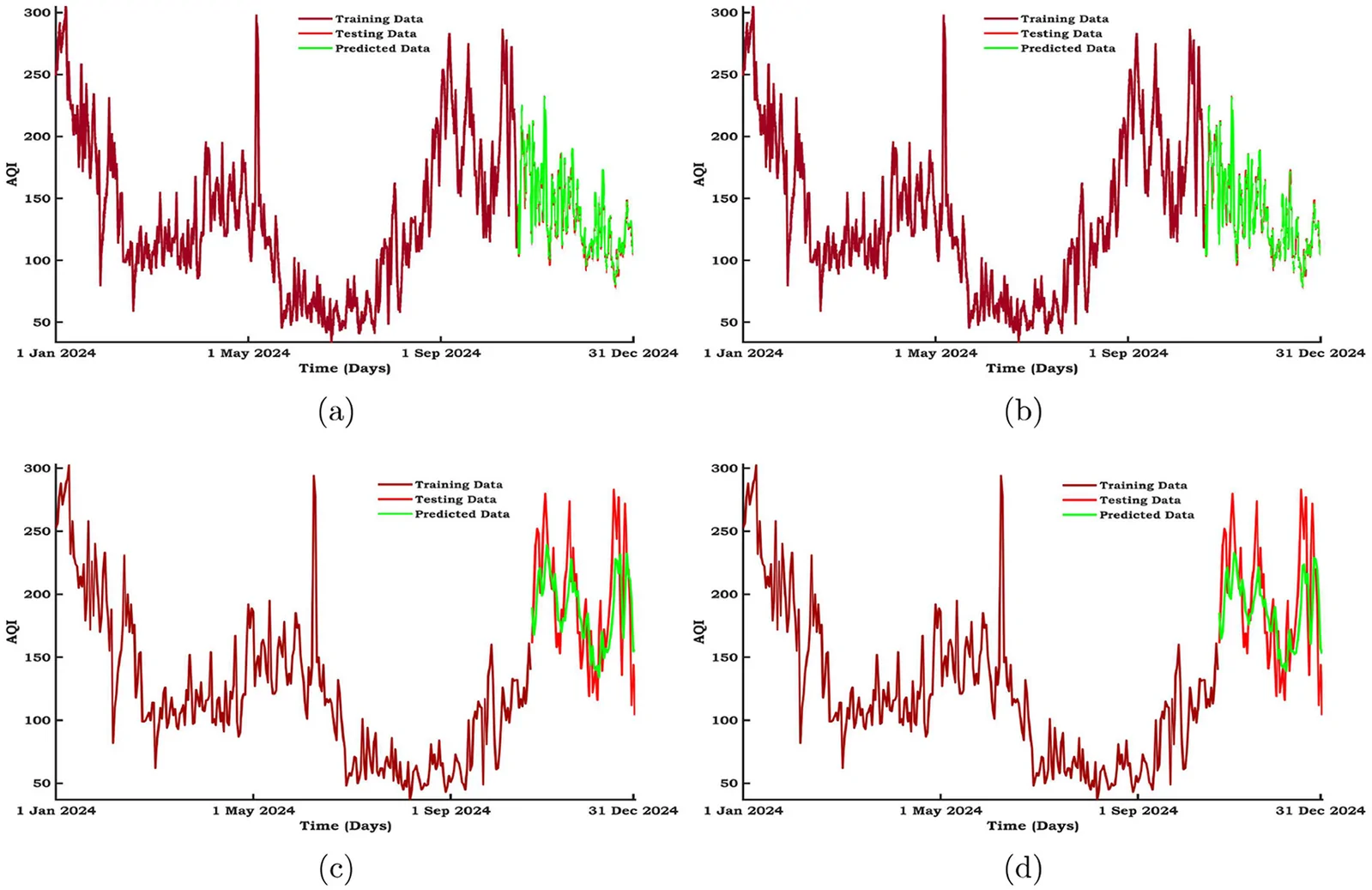
Comparison of AQI data associated with Jaipur after & before fractal pre-processing: **(a)** F-LSTM, **(b)** F-BiLSTM, **(c)** LSTM, **(d)** BiLSTM.

**Figure 12:**
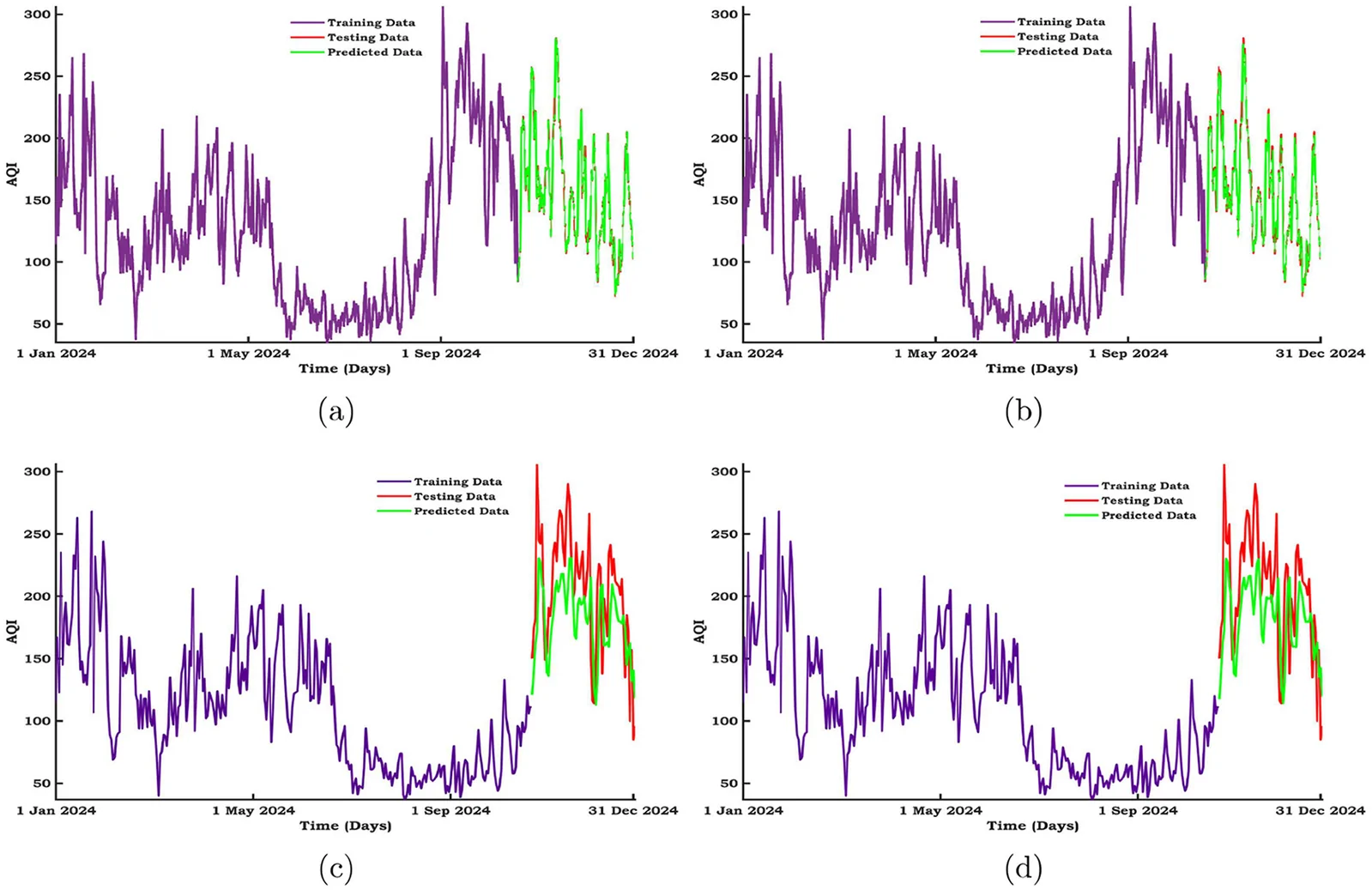
Comparison of AQI data associated with Lucknow after & before fractal pre-processing: **(a)** F-LSTM, **(b)** F-BiLSTM, **(c)** LSTM, **(d)** BiLSTM.

The pre-processed AQI data of the chosen five cities is trained and tested using LSTM, BiLSTM, and the models using fractal pre-processed data are represented using F-LSTM, F-BiLSTM, and F-CNN-LSTM. For comparison reasons, the non-pre-processed original AQI data are also trained and tested using deep learning models, and both the results are illustrated in Figures 8–12. Figure 8 demonstrates the forecasting results of AQI data associated with the city of Delhi. Figures 8a, b show the prediction of pre-processed data using LSTM and BiLSTM, respectively. Figures 8c, d demonstrate the prediction of original data using LSTM and BiLSTM, respectively. The orange curve indicates the trained AQI data, the red curve indicates the tested AQI data, and the green curve indicates the predicted AQI data of Delhi. The prediction results of Faridabad AQI before and after pre-processing are illustrated in Figures 9a–d, respectively. Similarly, the prediction results of AQI data associated with Bhopal, Jaipur, and Lucknow before and after pre-processing are presented in Figures 10–12, respectively.

While comparing the Figures 8a, b with the Figures 8–12c, d, predicted data fits the tested data much better in the case of fractal pre-processing than in the case of original AQI data. The better fitting is achieved due to the advantage of fractal functions in generating a large set of data, though a smaller number of sample data is available. The performance of LSTM and BiLSTM of pre-processed AQI is assessed using the evaluation metrics RMSE, NRMSE, MAE, MAPE, NMAPE, and R^2^. The obtained metric values associated with F-LSTM, F-BiLSTM, and F-CNN-LSTM of five cities are presented in Tables 4–6, respectively. In this context, tabulated findings of statistical evaluation reveal that the deep learning models demonstrate strong predictive performance despite the discrepancies in the original AQI data.

**Table 4:** Evaluation metric results of F-LSTM network.

Metric	Delhi	Faridabad	Bhopal	Jaipur	Lucknow
RMSE	1.9926	0.8312	0.9910	1.5249	0.8271
NRMSE	0.0063	0.0036	0.0059	0.0098	0.0039
MAE	1.7162	0.6204	0.7409	1.3889	0.6211
MAPE	0.7284	0.5184	0.5777	1.0811	0.4327
NMAPE	0.7423	0.4551	0.5982	1.0129	0.3952
*R* ^2^	0.9993	0.9997	0.9978	0.9976	0.9996

**Table 5:** Evaluation metric results of F-BiLSTM network.

Metric	Delhi	Faridabad	Bhopal	Jaipur	Lucknow
RMSE	1.7068	1.8153	1.0649	0.7558	1.5809
NRMSE	0.0054	0.0078	0.0064	0.0048	0.0076
MAE	1.4154	1.5619	0.8366	0.5706	1.2419
MAPE	0.7705	1.4879	0.6686	0.4304	0.7595
NMAPE	0.6122	1.1452	0.6754	0.4162	0.7902
*R* ^2^	0.9995	0.9988	0.9976	0.9994	0.9987

**Table 6:** Evaluation metric results of F-CNN-LSTM network.

Metric	Delhi	Faridabad	Bhopal	Jaipur	Lucknow
RMSE	1.0142	0.9305	1.2904	1.0224	0.8547
NRMSE	0.0032	0.0040	0.0078	0.0066	0.0041
MAE	0.8011	.06965	1.1018	0.8196	0.6329
MAPE	0.4000	0.5829	0.9146	0.6506	0.4385
NMAPE	0.3465	0.5109	0.8895	0.5977	0.4027
*R* ^2^	0.9998	0.9996	0.9964	0.9989	0.9996

**Figure 13:**
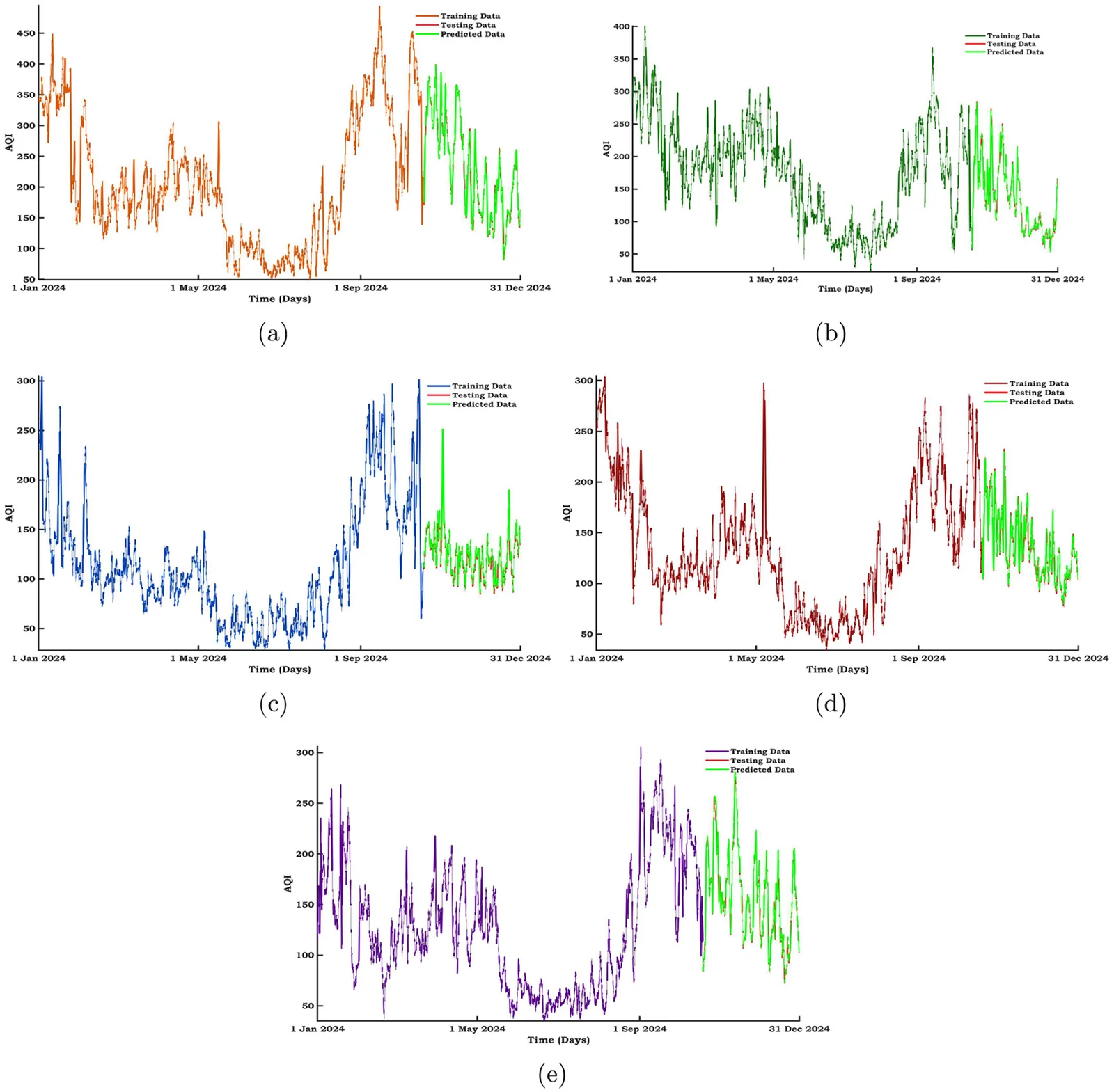
Comparison of CNN-LSTM based AQI data prediction associated with fractal pre-processing: **(a)** Delhi, **(b)** Faridabad, **(c)** Bhopal, **(d)** Jaipur, **(e)** Lucknow.

The prediction results of pre-processed AQI data using F-CNN-LSTM are illustrated in Figure 13. The AQI data performances for five cities—Delhi, Faridabad, Bhopal, Jaipur, and Lucknow—are shown in Figures 13a–e, respectively. This figure illustrates how well the predicted data matches the tested data. Additionally, the F-CNN-LSTM performance is validated by estimating the error evaluation metrics. Table 6 contains a tabulation of the evaluation outcomes. The NRMSE, MAPE, NMAPE, and *R*^2^ associated with Delhi have the most appropriate values of 0.0032, 0.4000, 0.3465, and 0.9998 compared to other cities, even if all the metrics show beneficial values for all the cities.

**Figure 14:**
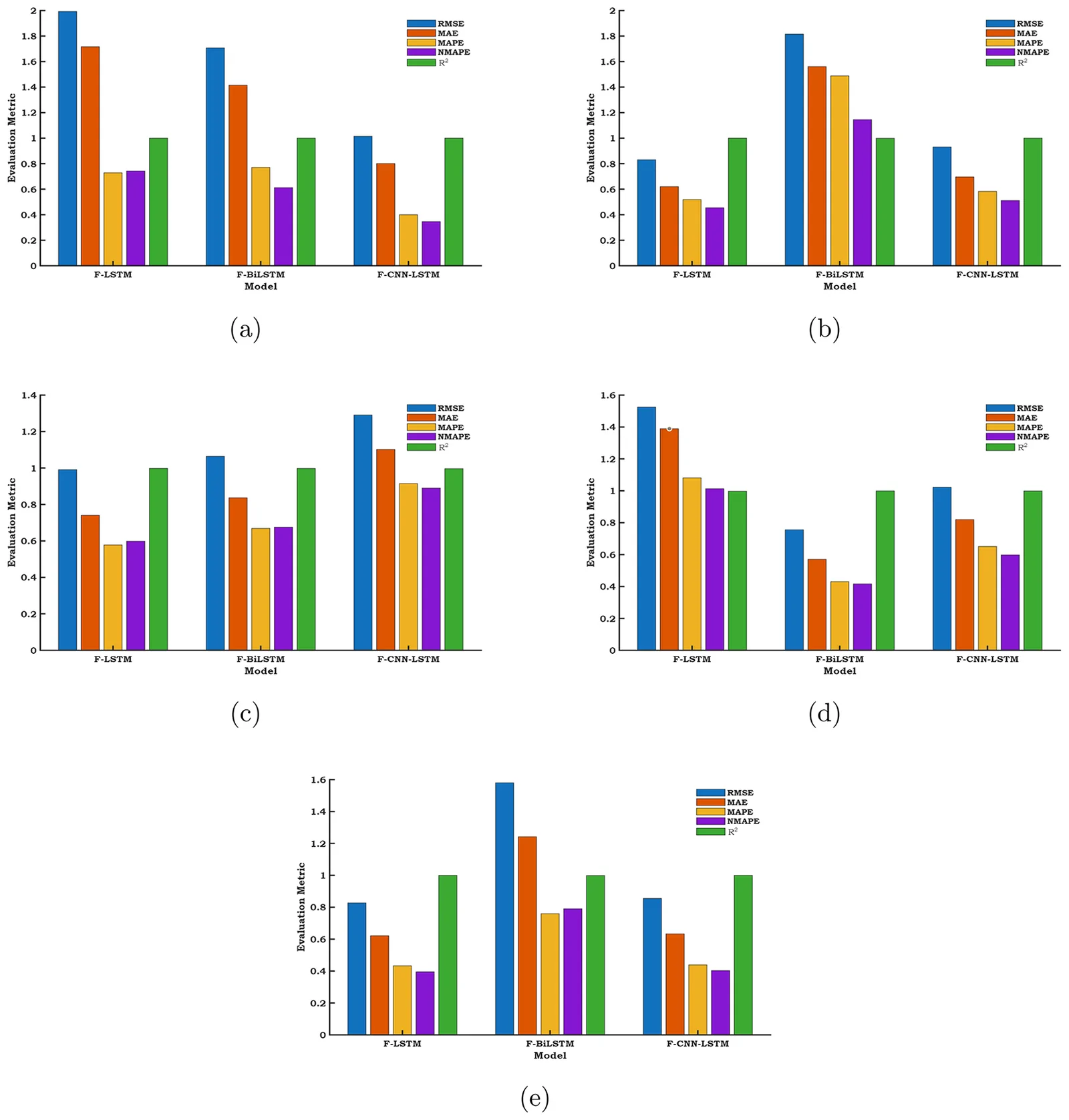
Comparison of box-whisker plot for AQI prediction error: **(a)** Delhi, **(b)** Faridabad, **(c)** Bhopal, **(d)** Jaipur, **(e)** Lucknow.

**Figure 15:**
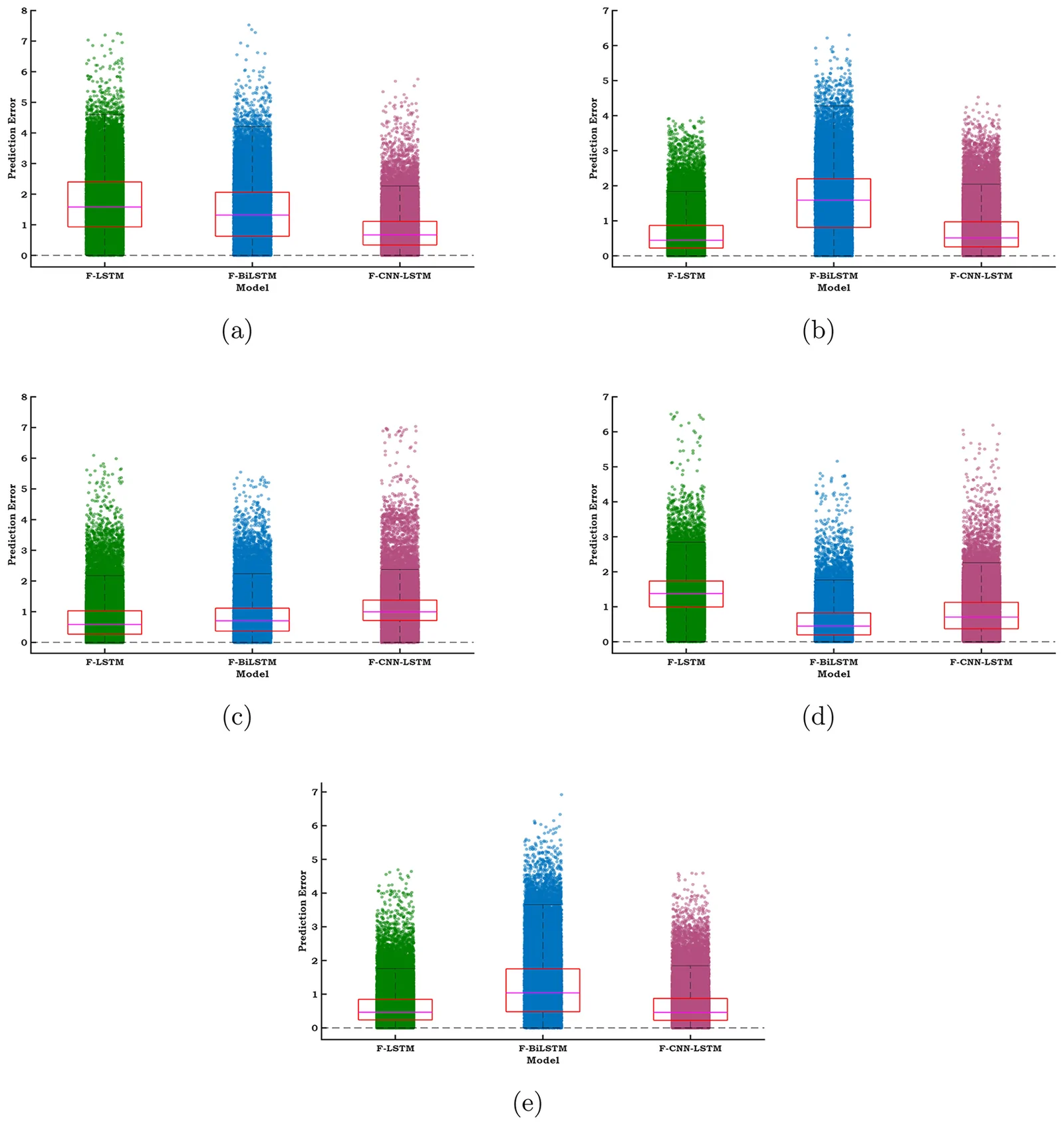
Performance analysis of three models, F-LSTM, F-BiLSTM, and F-CNN-LSTM using evaluation metrics for AQI prediction error: **(a)** Delhi, **(b)** Faridabad, **(c)** Bhopal, **(d)** Jaipur, **(e)** Lucknow.

Additionally, box-whisker plots are used to provide a prediction comparison of all three types of fractal integrated deep learning models. A useful technique for visually depicting the data distribution through its quartiles is the box-whisker plot. The lines that run parallel to the boxes are called “whiskers,”, and they are used to illustrate variability outside of the upper and lower quartiles. The box-whisker graphs in Figure 14 show the distribution of prediction errors for five cities using three models: F-LSTM, F-BiLSTM, and F-CNN-LSTM. While F-LSTM achieves smaller errors than the other two models in the Faridabad AQI data, which displays larger dispersion, F-CNN-LSTM demonstrates lower median errors for the Delhi AQI data, indicating steady and accurate predictions. Both the F-LSTM and F-BiLSTM graphs show a smaller spread in the Bhopal AQI forecast. For the AQI data from Jaipur and Lucknow, the median errors of F-BiLSTM and F-LSTM, respectively, are lower. Figure 15 illustrates the bar chart representations of tabulated statistical metric values provided in Tables 4–6. From this figure, for Delhi AQI data, the F-CNN-LSTM model outperforms F-LSTM and F-BiLSTM in terms of overall forecasting performance and prediction errors. For Faridabad AQI forecasting, the F-LSTM model surpasses the other two models in terms of predicted accuracy and error levels. The F-BiLSTM model provides improved prediction performance compared to F-LSTM and F-CNN-LSTM for Bhopal AQI data. The best AQI prediction performance for Jaipur data is demonstrated by the F-BiLSTM model, which has the lowest forecasting errors. For the Lucknow AQI predictions, the F-LSTM model is superior to the other models in terms of predictive stability and error metrics. This improved accuracy is attributed to the fractal-based pre-processing, which contributed to enhancing data structure and capturing the underlying complexity, convincingly illustrating the study's goal.

**Figure 16:**
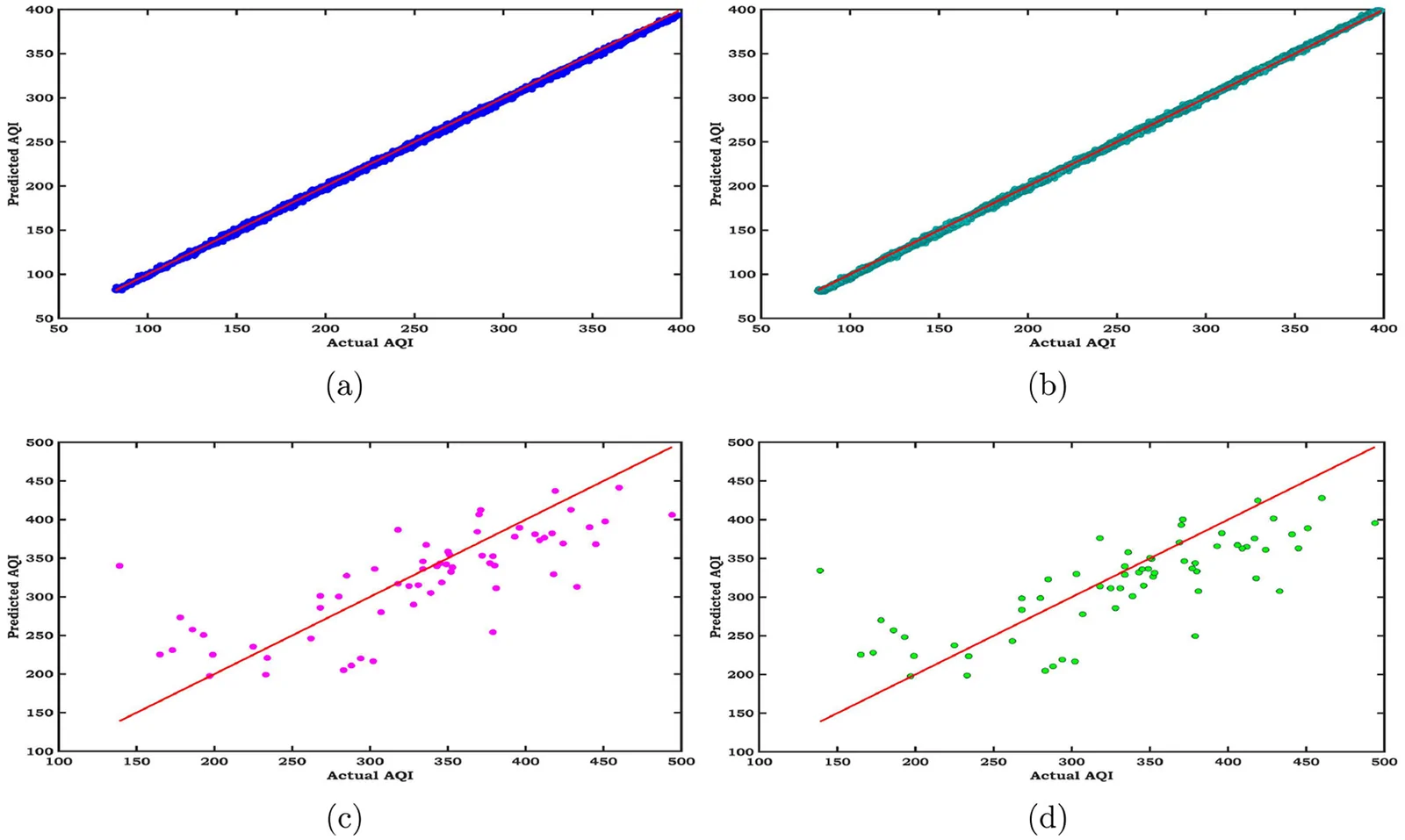
Actual AQI vs. predicted AQI scatter plots of Delhi: **(a)** F-LSTM, **(b)** F-BiLSTM, **(c)** LSTM, **(d)** BiLSTM.

**Figure 17:**
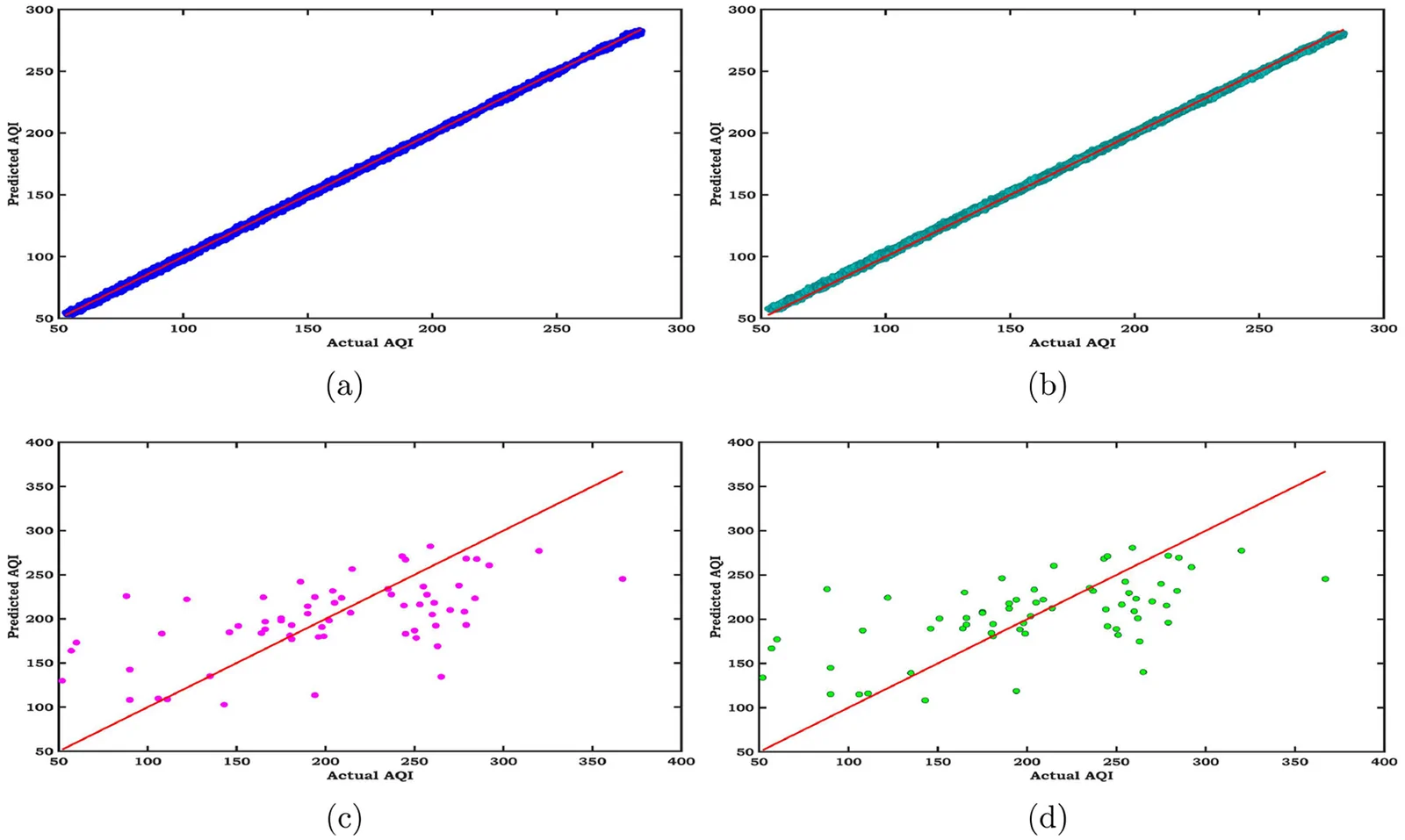
Actual AQI vs. predicted AQI scatter plots of Faridabad: **(a)** F-LSTM, **(b)** F-BiLSTM, **(c)** LSTM, **(d)** BiLSTM.

**Figure 18:**
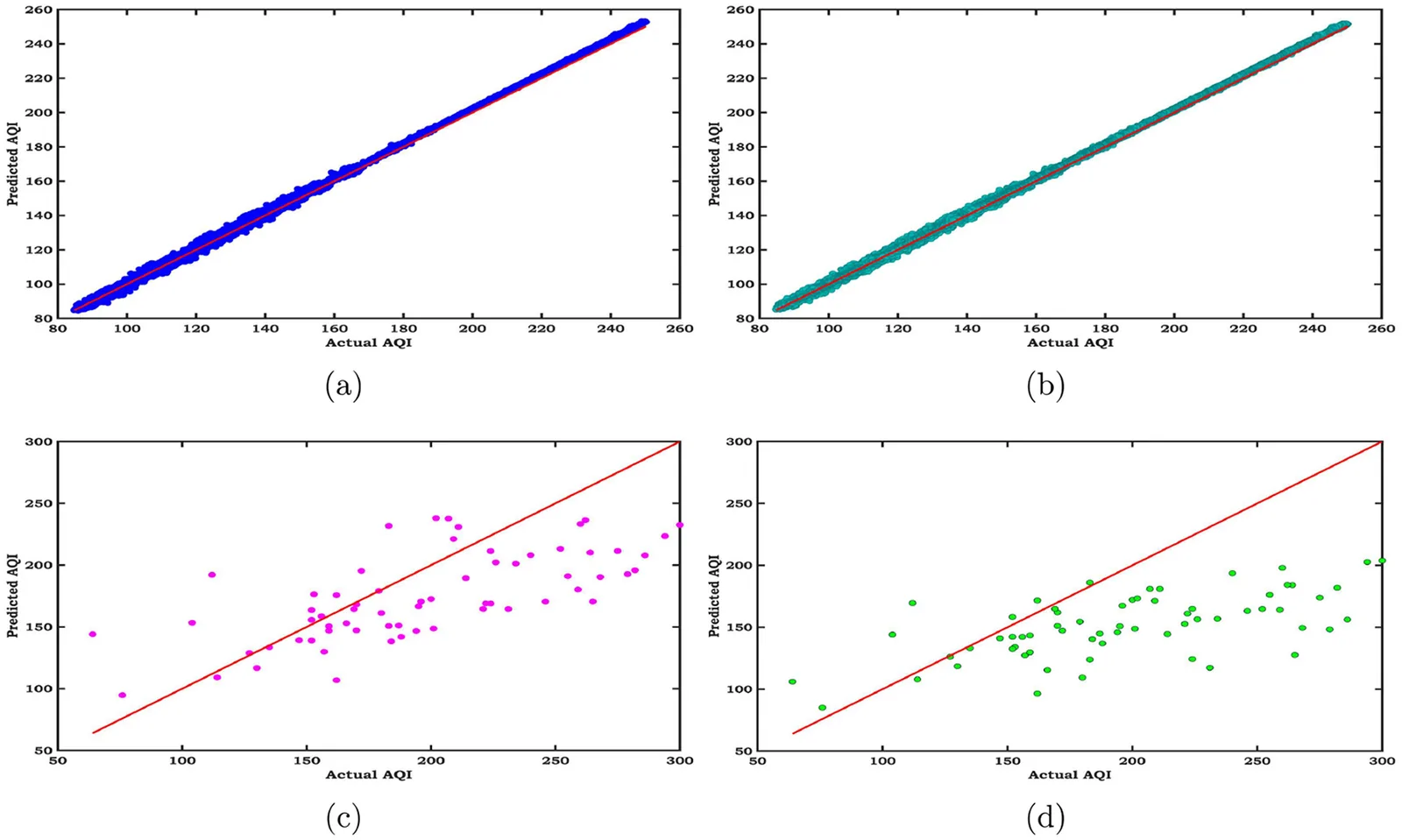
Actual AQI vs. predicted AQI scatter plots of Bhopal: **(a)** F-LSTM, **(b)** F-BiLSTM, **(c)** LSTM, **(d)** BiLSTM.

**Figure 19:**
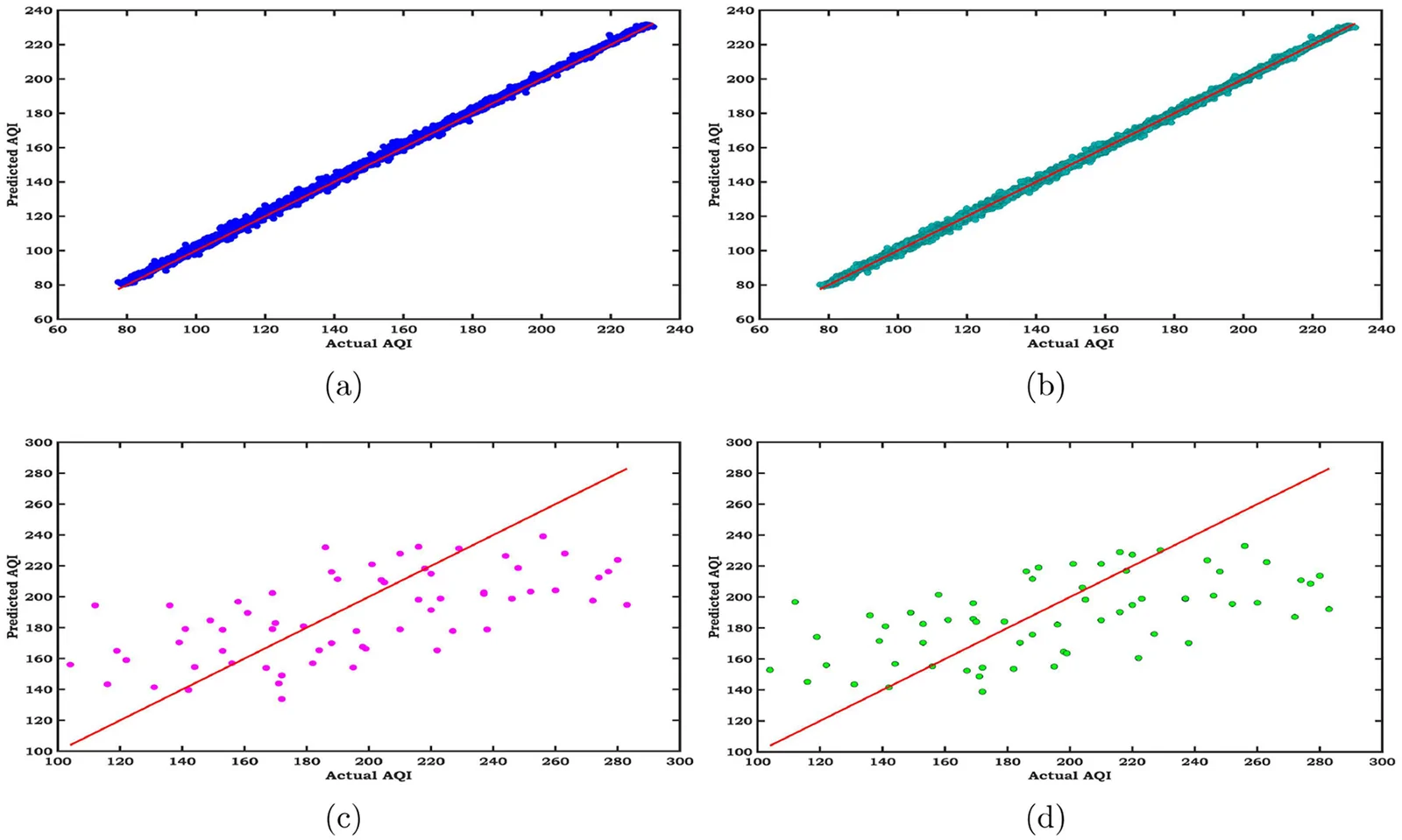
Actual AQI vs. predicted AQI scatter plots of Jaipur: **(a)** F-LSTM, **(b)** F-BiLSTM, **(c)** LSTM, **(d)** BiLSTM.

**Figure 20:**
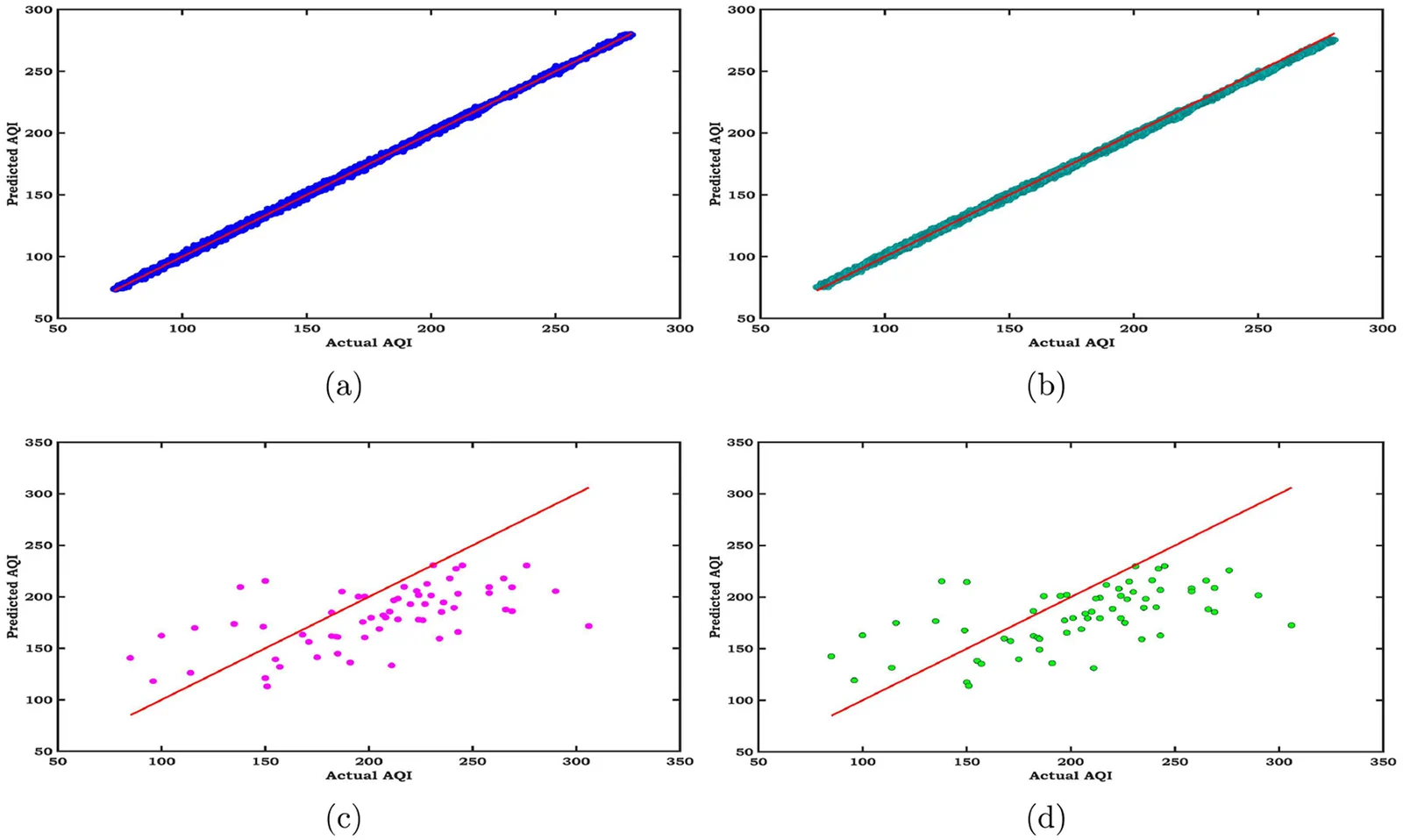
Actual AQI vs. predicted AQI scatter plots of Lucknow: **(a)** F-LSTM, **(b)** F-BiLSTM, **(c)** LSTM, **(d)** BiLSTM.

Further, scatter plot visualizations are illustrated in Figures 16–20 to compare the distribution and data range of actual and predicted AQI values. The figures demonstrate the areas of optimal performance for the employed techniques. The actual AQI values are represented by the horizontal axis, whereas the AQI predictions are represented by the vertical axis. The graphs show that, compared to the LSTM and BiLSTM models, the F-LSTM and F-BiLSTM models obtain a very close alignment between the actual and predicted AQI values. In fact, the current study compares standard deep learning models (LSTM and BiLSTM) with their fractal-enhanced counterparts (F-LSTM and F-BiLSTM) in an ablation-style approach. The purpose of this comparative framework was to identify and assess the contribution of the suggested fractal pre-processing method. The impact of fractal-based data enrichment on AQI forecasting can be directly examined by contrasting the prediction performances before and after using fractal interpolation. The scatter plots in Figures 16–20 provide the comparison analysis as an ablation study of prediction results with and without fractal pre-processing. In comparison to the equivalent baseline models, the acquired results consistently show that the fractal-enhanced models produce decreased prediction errors and increased accuracy metrics.

### 4.4 Wilcoxon test analysis

A nonparametric statistical test called the Wilcoxon signed-rank test is used to evaluate two related models and assess whether there is a statistically significant difference in their performances. The null hypothesis (*H*_0_) in this test asserts that there is no significant difference between the paired observations, while the alternative hypothesis (*H*_1_) asserts that there is a significant difference. The p-values of the test are used to assess the significance of the results. If the p-value is less than the selected significance level (usually 0.05), then the alternative hypothesis is accepted, and the null hypothesis is rejected, suggesting statistically significant differences between the examined models.

From Table 7, based on statistical tests and error analysis, the models show significant differences. The Mean Absolute Error (MAE) values obtained for the three models are as follows: F-LSTM = 1.7162, F-BiLSTM = 1.4154, F-CNN-LSTM = 0.8011. Based on the MAE values, the ranking of the models is as follows: F-CNN-LSTM, F-BiLSTM, and F-LSTM. The Wilcoxon signed-rank test produced *p*-values less than 0.05 for all pairwise comparisons, indicating that the performance differences between the models are statistically significant. From Table 7, it can be concluded that the F-CNN-LSTM model has achieved the best forecasting performance with the lowest prediction error in Delhi's AQI data, followed by the F-BiLSTM and F-LSTM models.

**Table 7:** Wilcoxon pairwise comparison for Delhi AQI data.

Comparison	*P*-Value	*H*_1_ status	Significance
F-LSTM vs. F-BiLSTM	0	Accepted	Significant
F-LSTM vs. F-CNN-LSTM	0	Accepted	Significant
F-BiLSTM vs. F-CNN-LSTM	0	Accepted	Significant

Table 8 provides the Wilcoxon pairwise comparison for Faridabad AQI data. With p-values less than 0.05 for all pairwise comparisons, the Wilcoxon signed-rank test demonstrate the statistical significance in performance differences. MAE values obtained for the three models are as follows: F-LSTM = 0.6204, F-BiLSTM = 1.5610, F-CNN-LSTM = 0.6965. Based on the MAE values, the ranking of the models is as follows: F-LSTM, F-CNN-LSTM, and F-BiLSTM. Among the three models, F-LSTM model achieved the best forecasting performance with the lowest prediction error in Faridabad's AQI data, followed by the F-BiLSTM and F-CNN-LSTM models.

**Table 8:** Wilcoxon pairwise comparison for Faridabad AQI data.

Comparison	*P*-Value	*H*_1_ status	Significance
F-LSTM vs. F-BiLSTM	0	Accepted	Significant
F-LSTM vs. F-CNN-LSTM	0	Accepted	Significant
F-BiLSTM vs. F-CNN-LSTM	0	Accepted	Significant

In Table 9, the Wilcoxon signed-rank test produced p-values less than 0.05 for all three different pairwise comparisons, confirming the statistical significance in the performance differences among the three models. MAE values of F-LSTM, F-BiLSTM, and F-CNN-LSTM are 0.7410, 0.8366, and 1.1018, respectively. Based on the MAE values, the ranking of the models is as follows: F-LSTM, F-BiLSTM, F-CNN-LSTM. Hence, it can be reported that for the Bhopal's AQI data, the F-LSTM model achieved the best forecasting performance with the lowest prediction error, followed by the F-BiLSTM and F-CNN-LSTM models.

**Table 9:** Wilcoxon pairwise comparison for Bhopal AQI data.

Comparison	*P*-Value	*H*_1_ status	Significance
F-LSTM vs. F-BiLSTM	0	Accepted	Significant
F-LSTM vs. F-CNN-LSTM	0	Accepted	Significant
F-BiLSTM vs. F-CNN-LSTM	0	Accepted	Significant

The Wilcoxon signed-rank test in Table 10 shows statistically significant differences in the models' performance with *p*-values less than 0.05. The three models' MAE values are as follows: F-LSTM = 1.3889, F-BiLSTM = 0.5706, and F-CNN-LSTM = 0.8196. The models are ranked as follows based on the MAE values: F-BiLSTM, F-CNN-LSTM, and F-LSTM. Therefore, with Jaipur's AQI data, the F-BiLSTM model produced the best forecasting performance with the lowest prediction error, followed by the F-LSTM and F-CNN-LSTM models.

**Table 10:** Wilcoxon pairwise comparison for Jaipur AQI data.

Model comparison	*P*-Value	*H*_1_ status	Significance
F-LSTM vs. F-BiLSTM	0	Accepted	Significant
F-LSTM vs. F-CNN-LSTM	0	Accepted	Significant
F-BiLSTM vs. F-CNN-LSTM	0	Accepted	Significant

Table 11 shows that the Wilcoxon signed-rank test produced *p*-values less than 0.05 for all pairwise comparisons, indicating statistically significant differences in the model's performance. MAE values obtained for F-LSTM, F-BiLSTM, and F-CNN-LSTM are 0.6212, 1.2419, and 0.6330, respectively. Based on the MAE values, the ranking of the models is as follows: F-LSTM, F-CNN-LSTM, and F-BiLSTM. Hence, the F-LSTM model has achieved the best forecasting performance with the lowest prediction error in Lucknow's AQI data, followed by the F-BiLSTM and F-CNN-LSTM models.

**Table 11:** Wilcoxon pairwise comparison for Lucknow AQI data.

Model comparison	*P*-Value	*H*_1_ status	Significance
F-LSTM vs. F-BiLSTM	0	Accepted	Significant
F-LSTM vs. F-CNN-LSTM	0	Accepted	Significant
F-BiLSTM vs. F-CNN-LSTM	0	Accepted	Significant

## 5 Limitations

Despite showing promising outcomes for AQI prediction, the suggested fractal-integrated deep learning system has some limitations that should be noted. The quality, accessibility, and temporal features of the air quality datasets, as well as the choice of suitable fractal scaling factors, may have an impact on the forecast accuracy. Furthermore, since the current study concentrates on five extremely polluted Indian cities, additional validation utilizing various geographic locations and environmental circumstances is necessary to show wider applicability. By adding more influencing factors, such as climatic variables, and combining sophisticated optimization techniques for adaptive fractal parameter selection, the suggested framework can be expanded. The robustness and transferability of the suggested method for extensive air quality forecasting applications may be further improved by these extensions.

## 6 Conclusion

Public health, industrial sustainability, and rapid responses to environmental risks - which are crucial for sustainable development - are all greatly impacted by air quality. Therefore, accurate air quality index predictions are significant for efficient environmental management and tracking. This study has proposed three integrated fractal-deep learning models, F-LSTM, F-BiLSTM, and CNN-LSTM for AQI prediction in very air-polluted cities of India, namely, Delhi, Faridabad, Bhopal, Jaipur, and Lucknow. The purpose of integrating fractal with deep learning is clarified with the process of data pre-processing. The standalone performances of F-LSTM, F-BiLSTM, and CNN-LSTM are compared using RMSE, NRMSE, MAE, MAPE, NMAPE, and the findings show a high coefficient of determination of *R*^2^. With the superior performance, the proposed integrated models can also be extended by combining with machine learning models like gated recurrent units as a future direction. In the aspect of fractal technique, different fractal interpolation functions with different optimized scaling formulae can be used for better results. Further, due to the advantage of large data set generation, the fractal technique can be used to enlarge any real small data set and utilized with any hybrid models for more effectiveness in AQI prediction.

In the proposed fractal-integrated framework, the vertical scaling parameters are chosen to meet the contractive requirement |α_*k*_| < 1. These scaling factors are selected within the allowable range using standard fractal interpolation limitations. Even if the results show how superior the proposed method is, a better choice of vertical scaling factors could improve prediction performance even further. Thus, optimization-based techniques to find appropriate scaling parameters that meet |α_*k*_| < 1 can be developed as a future research path, while reducing interpolation and prediction errors, enhancing the suggested AQI forecasting framework's flexibility and resilience.

## Data Availability

The original contributions presented in the study are included in the article/supplementary material, further inquiries can be directed to the corresponding author.
